# The bacterial community significantly promotes cast iron corrosion in reclaimed wastewater distribution systems

**DOI:** 10.1186/s40168-018-0610-5

**Published:** 2018-12-13

**Authors:** Guijuan Zhang, Bing Li, Jie Liu, Mingqiang Luan, Long Yue, Xiao-Tao Jiang, Ke Yu, Yuntao Guan

**Affiliations:** 1 Guangdong Provincial Engineering Research Center for Urban Water Recycling and Environmental Safety, Graduate School at Shenzhen, Tsinghua University, Shenzhen, China; 2 State Environmental Protection Key Laboratory of Microorganism Application and Risk Control, School of Environment, Tsinghua University, Beijing, China; 3 Microbiome Research Centre, St George and Sutherland Clinical School, Department of Medicine, University of New South Wales, Sydney, Australia; 4 School of Environment and Energy, Shenzhen Graduate School, Peking University, Shenzhen, China

**Keywords:** Corrosion, Reclaimed wastewater, High-throughput sequencing, Bacterial community, *Desulfovibrio*

## Abstract

**Background:**

Currently, the effect of the bacterial community on cast iron corrosion process does not reach consensus. Moreover, some studies have produced contrasting results, suggesting that bacteria can either accelerate or inhibit corrosion.

**Results:**

The long-term effects of the bacterial community on cast iron corrosion in reclaimed wastewater distribution systems were investigated from both spatial (yellow layer vs. black layer) and temporal (1-year dynamic process) dimensions of the iron coupon-reclaimed wastewater microcosm using high-throughput sequencing and flow cytometry approaches. Cast iron coupons in the NON_disinfection_ and UV_disinfection_ reactors suffered more severe corrosion than did those in the NaClO_disinfection_ reactor. The bacterial community significantly promoted cast iron corrosion, which was quantified for the first time in the practical reclaimed wastewater and found to account for at least 30.5% ± 9.7% of the total weight loss. The partition of yellow and black layers of cast iron corrosion provided more accurate information on morphology and crystal structures for corrosion scales. The black layer was dense, and the particles looked fusiform, while the yellow layer was loose, and the particles were ellipse or spherical. Goethite was the predominant crystalline phase in black layers, while corrosion products mainly existed as an amorphous phase in yellow layers. The bacterial community compositions of black layers were distinctly separated from yellow layers regardless of disinfection methods. The NON_disinfection_ and UV_disinfection_ reactors had a more similar microbial composition and variation tendency for the same layer type than did the NaClO_disinfection_ reactor. Biofilm development can be divided into the initial start-up stage, mid-term development stage, and terminal stable stage. In total, 12 potential functional genera were selected to establish a cycle model for Fe, N, and S metabolism. *Desulfovibrio* was considered to accelerate the transfer of Fe^0^ to Fe^2+^ and speed up weight loss.

**Conclusion:**

The long-term effect of disinfection processes on corrosion behaviors of cast iron in reclaimed wastewater distribution systems and the hidden mechanisms were deciphered for the first time. This study established a cycle model for Fe, N, and S metabolism that involved 12 functional genera and discovered the significant contribution of *Desulfovibrio* in promoting corrosion.

**Electronic supplementary material:**

The online version of this article (10.1186/s40168-018-0610-5) contains supplementary material, which is available to authorized users.

## Background

Wastewater reclamation and reuse is an effective way to relieve the dilemma of water resource shortages. Reclaimed wastewater can be used for irrigation, industrial consumption, and supplementation of ecological water, i.e., artificial wetlands and rivers. Cast iron pipes have been widely used in water distribution systems for more than 150 years because of their high mechanical strength and cost effectiveness [1]. In contrast to the corrosion of drinking water distribution systems (DWDS), which has attracted the attention of many researchers due to its serious effect of “red water” or “colored water” on people’s daily life [2–7], only a few studies have investigated corrosion in reclaimed wastewater distribution systems (RWDS). Reclaimed wastewater contains a much higher concentration of organic matter than does drinking water, which could result in the consumption of more disinfectants and promote the regrowth of more abundant and diverse bacteria [8]. The above features of reclaimed wastewater might lead to more severe corrosion of cast iron pipes and to consequent pipeline burst, resulting in loss of reclaimed wastewater. Additionally, considering scientific issues, the corrosion mechanism in RWDS may be strikingly different from that in DWDS. Corrosion is a synergistic interaction among the metal surface, abiotic corrosion products, bacterial cells, and their metabolites [9]. It can be affected by various factors, such as water quality, disinfection method, and microbial community structure [10]. Alkalinity and calcium hardness have been found to inhibit corrosion [7]. Among disinfectants, it is generally accepted that sodium hypochlorite and its residuals increase corrosion rates [11, 12].

Currently, the effect of microorganisms on the cast iron corrosion process has not reached consensus. Some studies have produced contrasting results, suggesting that microorganisms can either accelerate or inhibit corrosion [3]. In general, the main bacterial species related to metal transformation in terrestrial and aquatic habitats are sulfate-reducing bacteria (SRBs), sulfur-oxidizing bacteria (SOBs), iron-oxidizing bacteria (IOBs), and iron-reducing bacteria (IRBs) [13]. Numerous studies have investigated the impact of pure or artificially mixed culture bacteria on cast iron corrosion in water distribution pipelines. Sulfate-reducing bacteria are usually related to anaerobic iron corrosion [14], and artificially mixed cultures verified that the promotion of corrosion by SRBs can be diminished in the presence of *Pseudomonas aeruginosa* (denitrifying bacterium) [15]. Sulfur-oxidizing bacteria are believed to accelerate corrosion because of their ability to produce acid [16]. The presence of IOBs rapidly inhibited corrosion on cast iron coupons due to the formation of a passive layer in the early stage (approximately the first 20 days) and accelerated corrosion with the decrease in passive layer adhesion [4]. Iron-reducing bacteria can enhance corrosion by the reduction of Fe^3+^ corrosion products, which are easily dissolved to expose the metal surface to the corrosive medium again. However, IRBs can also inhibit corrosion by developing biofilms at the metal surface and producing the extracellular polymeric substance (EPS) as a protective layer [17]. It should be noted that in natural environments and engineered systems, microbial biofilms are always composed of multifarious bacteria and not merely a single bacterium or several types of bacteria. Therefore, the effect of the microbial community on cast iron corrosion has attracted increasing attention. Some researchers believe that the effect of biofilm on cast iron corrosion in RWDS changes over time. In a 30-day experiment, Teng et al. [18] verified that biofilm accelerated corrosion within 7 days but inhibited corrosion after 7 days. The major reason for this result was the abundance transition of IOBs and IRBs. Wang et al. [12] considered that corrosion-inducing bacteria, including the IRB *Shewanella sp.*, the IOB *Sediminibacterium sp.*, and the SOB *Limnobacter thiooxidans*, promoted iron corrosion by synergistic interactions in the primary period. Nevertheless, when IRBs became the dominant bacteria, they could prevent further corrosion via the formation of protective layers. Some studies have demonstrated that the existence of biofilm in reclaimed wastewater significantly promoted corrosion [19]. However, it was suggested that biofilm could protect metal from corrosion by preventing the diffusion of oxygen [20]. Other researchers did not reach a clear conclusion but rather speculated that bacterial communities could at least promote the layering process and the formation of corrosion tubercles [8].

Whether microbes inhibit or promote corrosion, their remarkable effect on corrosion has been affirmed, especially the impact of anaerobic bacteria existing close to the base of cast iron. Nevertheless, previous studies did not distinguish the anaerobic layer from the aerobic layer when investigating the effect of the microbial community on cast iron corrosion in RWDS. Furthermore, no information is available on the relationship between the dynamics of the bacterial community composition of different layers and the corrosion behaviors in RWDS over time, especially in the long term. With the rapid development of high-throughput sequencing technology, the dynamics of microbial community structure and its effect on corrosion could be disclosed with high resolution and high accuracy [21].

Considering the above research gaps, the questions that we wish to address in this study are summarized as follows. (i) Do different disinfection processes (NaClO_disinfection_ and UV_disinfection_) affect the corrosion behaviors of cast iron in reclaimed wastewater in the long term compared to nondisinfection process (NON_disinfection_)? (ii) Does the bacterial community promote or inhibit corrosion of cast iron? (iii) Are there some key bacterial species contributing to corrosion inhibition or promotion? (iv) How do functional microorganisms drive the iron element cycle in the interface microcosm of cast iron-reclaimed wastewater?

## Methods

### Laboratory-scale reactor setup

Three laboratory-scale reactors were set up to simulate RWDS (Additional file 1: Figure S1) and were placed in the dark to prevent the growth of phototrophic microorganisms at the Xili reclaimed wastewater plant (RWP) in Shenzhen, Guangdong Province, China. Xili RWP uses a BIOSTYR® biological active filter and an ACTIFLO® high-density settling basin (Veolia Water, France) as the main treatment process with a treatment capacity of 50,000 m^3^/d. NaClO_disinfection_, UV_disinfection_ and NON_disinfection_ reclaimed wastewaters were pumped into the three reactors to compare the effect of disinfecting methods on the corrosion of cast iron coupons. Both NON_disinfection_ and NaClO_disinfection_ reclaimed wastewaters were collected from the secondary sedimentation tank effluent, and the latter was obtained by adding sodium hypochlorite (NaClO) with 5 mg/L free chlorine. The UV_disinfection_ reclaimed wastewater was collected from the UV disinfection tank directly. Three types of reclaimed wastewater were pumped into three 1000 L storage tanks and then discharged horizontally through the pipeline system at a rate of 0.2 m/s [8]. Ductile cast iron coupons (QT450), with C (3.4 ~ 3.9%), Si (2.2 ~ 2.8%), Mn (< 0.5%), P (< 0.07%), S (< 0.03%), Mg (0.03 ~ 0.06%), and Re (0.02 ~ 0.04%) were used in this study. Prior to the experiment, the coupons were first rinsed with deionized water thrice, degreased with acetone, sterilized by immersion in 70% ethanol for 8 h, and then dried aseptically in a laminar flow cabinet. Finally, the coupons were exposed to UV light for 30 min before they were weighed [22]. All water quality parameters were measured according to the standard methods [23] (see Additional file 1: Text S1). The detailed water quality of NaClO_disinfection_, NON_disinfection_, and UV_disinfection_ reclaimed wastewaters is summarized in Additional file 1: Table S1.

### Sample collection and preparation

To investigate the diversity and dynamics of the bacterial community on cast iron coupons, samples were collected from three reactors weekly for the first month and then every 3 weeks for the next 11 months, with a total of 20 sampling times during the entire experimental period. Sampling time points are shown in Additional file 1: Table S2. All samples were transported to the laboratory within 2 h for subsequent pretreatment and analysis. All analyses were conducted within 24 h. To distinguish the effect of metabolic bacterial activity on corrosion under aerobic and anaerobic conditions, biofilms in cast iron coupons were divided into two layers according to their color (Additional file 1: Figure S2). The surface layer was aerobic and yellow, while the inner layer was anaerobic and black. To obtain sufficient biomass on different layers, four pieces of cast iron coupons were collected each time, the surface layer and inner layer of each piece were separately sampled, and the same layers were mixed together. The surface layer, namely, the yellow layer, was flushed slightly with ultrapure water and finally collected a total of 500 mL of suspension liquid. To detach bacteria from the inner layer, i.e., the black layer, the cast iron coupons on which the yellow layer had already been removed were treated by ultrasonic processing (42 kHz) three times for 5 min each, and a total of 500 mL of suspension liquid was obtained [24, 25]. The potential biases of bacterial viability and adenosine triphosphate (ATP) measurement resulting from ultrasonic processing were excluded based on the preliminary experiment (see Additional file 1: Text S2). Four milliliters of obtained suspension liquid was used for further adenosine triphosphate (ATP) measurement and flow cytometry cell counting, and the other 496 mL was used for DNA extraction. The corrosion rate was determined by the weight loss method [10, 22]. The corrosive cast iron coupons were lyophilized for 24 h and gently divided into yellow layer and black layer by a sterile metal spatula. The crystalline phase of the yellow layer and black layer was characterized using an X-ray powder diffractometer (XRD; RIGAKU D/max2500/PC, Japan). The micrograph of the cast iron corrosion scale was examined by scanning electron microscopy operating at 15.0 kV (SU8010, HITACHI, Japan). In addition, polarization curves were also measured by an electrochemical workstation (CHI750e, Chenhua, Shanghai, China).

### Adenosine triphosphate measurement

To allow the bacteria to be adequately released from the iron rust, 0.25 mL of 0.5 mm glass beads was added into the suspension liquid obtained above. After a 60 s × 3 vortex pretreatment, supernatant was collected via centrifugation for 2 min at 600*g* [26] for ATP measurement using the BacTiter-Glo™ reagent (Promega Corporation, Madison, USA) and a luminometer (SpectraMax i3, Molecular Devices, USA) [27]. The data were collected as relative light units and converted to ATP (nM) by a calibration curve established with a known rATP standard (Promega).

### Flow cytometry measurement

To count viable/dead bacteria simultaneously, bacterial suspensions (1 mL) were stained with 10 μL/mL SYBR Green I (1:100 dilution in DMSO; Invitrogen) and 6 μM propidium iodide, which only stains damaged bacteria, and incubated in the dark for 25 min at room temperature before measurement. If necessary, samples were diluted to lower than 2 × 10^5^ cells/mL by cell-free Milli-Q water before measurement. Flow cytometry measurement was performed using FACSCalibur (BD, USA), emitting at a fixed wavelength of 488 nm and volumetric counting hardware. The signals of SYBR Green I and propidium iodide were respectively collected in the FL1 channel (520 nm) and the FL3 channel (615 nm), all data were processed with BD CellQuest™ Pro, and electronic gating with the software was used to separate positive signals from noise [27, 28].

### DNA extraction, PCR amplification, and Illumina sequencing

Microbial biomass was harvested from suspension liquid using 0.22 μm nitrocellulose membrane filters (47 mm diameter, Millipore, Billerica, MA, USA) [29]. Genomic DNA from the biomass in the black layer and yellow layer was separately extracted using a FastDNA® SPIN Kit for soil (MP Biomedicals, France) following the manufacturer’s instructions. The concentration and purity of DNA were determined using a NanoDrop 2000 spectrophotometer (Thermo Fisher Scientific, USA). The extracted DNA was stored at − 20 °C for subsequent use. For PCR amplification, the hypervariable V4 region of the bacterial 16S rRNA gene was amplified using a forward primer (5′-TATGGTAATTGTGTGCCAGCMGCCGCGGTAA-3′) and reverse primer (5′-AGTCAGTCAGCCGGACTACHVGGGTWTCTAAT-3′). Barcode was added at the 5′ end of the forward and reverse primers to allow for sample multiplexing during sequencing [30], resulting in a fragment size of 333 bp that was sequenced in a paired ends fashion, with read length of 250 bp per read-mate. PCR solutions contained 25 μL of ExTaq™ premix (Takara, China), 2 μL of 10 μM forward and reverse primers, 1 μL of 20 ng/μL DNA, and 22 μL of RNA-free H_2_O. The thermocycling steps for PCR were set as follows: initial denaturation at 95 °C for 5 min; 28 cycles at 95 °C for 30 s, 55 °C for 30 s, and 72 °C for 1 min; and a final extension step at 72 °C for 5 min. PCR products were purified using the MiniBEST DNA Fragment Purification Kit Ver. 4.0 (Takara, Japan) and then visualized on an agarose gel. Purified PCR amplicons were quantified by NanoDrop 2000 and mixed to achieve equal mass concentrations for paired-end 250 bp sequencing on a HiSeq 2500 platform.

### Bioinformatics analyses

All the raw sequencing data of the 16S rRNA amplicons were processed in Mothur v. 1.39.5 [31]. Briefly, sequences were first demultiplexed, quality trimmed, aligned, and finally checked with chimera.uchime to remove chimeric sequences, following the standard pipeline in the Mothur manual. Then, the clear sequences were normalized by randomly extracting 40,000 clean sequences from each sample dataset to fairly compare all samples at the same sequencing depth [32]. Next, the normalized sequences from all samples were clustered into operational taxonomic units (OTUs) at an identity threshold of 97%, which approximately corresponds to the taxonomic levels of species for bacteria. OTUs with an abundance of less than 10 sequences were removed from the OTU table. Representative sequences of OTUs were extracted and submitted to the Ribosomal Database Project (RDP) Classifier for taxonomy annotation at an 80% threshold. The diversity index and evenness were calculated using PAST 3 [33]. The taxonomic dendrogram was visualized by Cytoscape 3.6.0 [34] to obtain an overall view of the bacterial community structure. All sample similarities and pairwise comparisons between different layers were computed as weighted UniFrac distances [35] and were visualized by principal coordinate analysis (PCoA) using “vegan” and “ggplot2” packages in R studio.

## Results and discussion

### Corrosion process and corrosion scale characterization under different disinfection conditions

#### Corrosion process monitoring

**Fig. 1:**
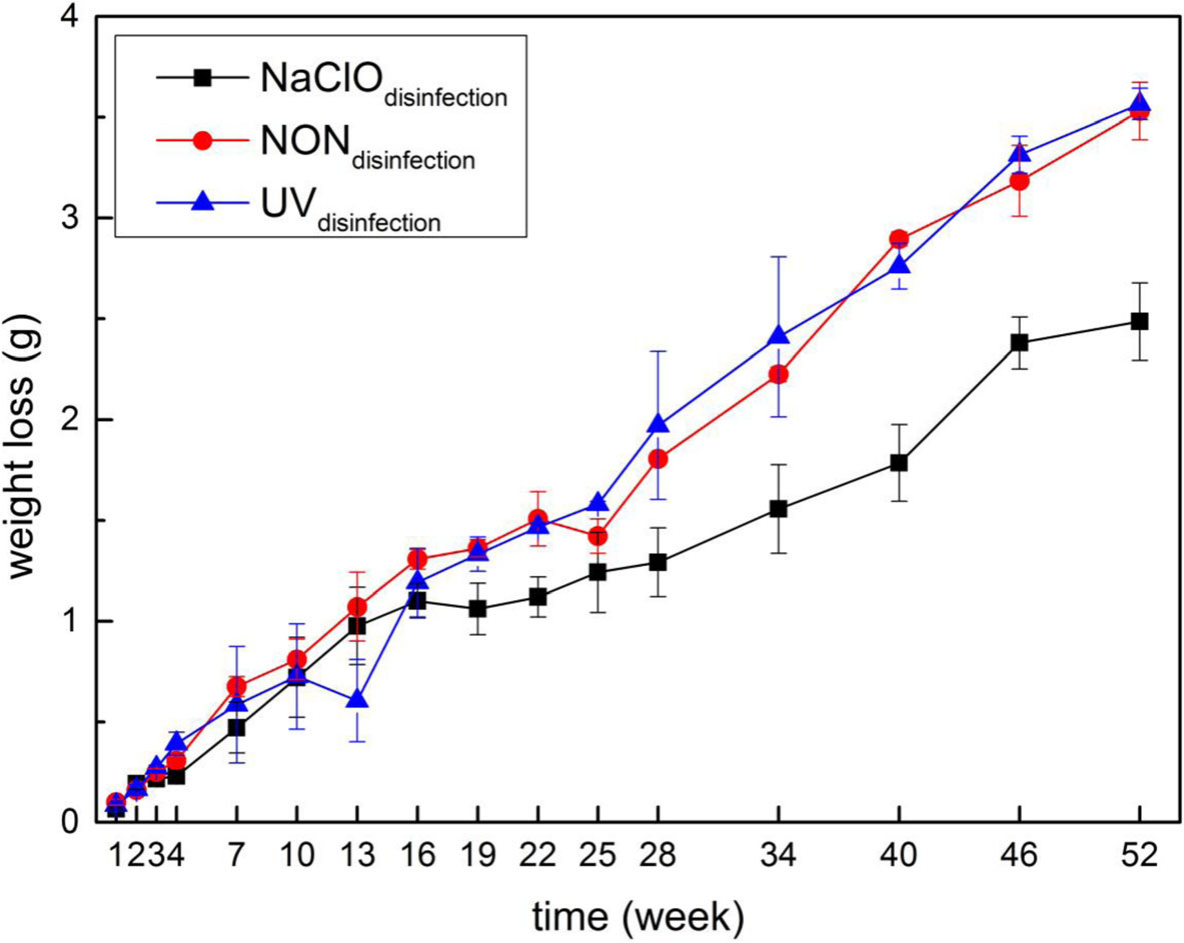
The weight loss of the cast iron coupons in the NaClO_disinfection_, NON_disinfection_, and UV_disinfection_ reactors during a 1-year period. Each data point represents the average weight loss of four pieces of cast iron coupons (*n* = 4). Error bars represent the standard deviation

The weight loss results indicated that the cast iron coupons in the NON_disinfection_ and UV_disinfection_ reactors suffered more severe corrosion than did those in the NaClO_disinfection_ reactor (Fig. 1), which seemed contradictory to the expectation because NaClO was thought to promote corrosion [11, 12]. The detailed explanation will be discussed in the subsection “Morphology and crystal structures of the corrosion scale.” Before the 19th week, the weight loss of the cast iron coupons in the three reactors did not significantly differ (*P* > 0.05, paired *t* test). After the 19th week, the weight loss of the cast iron coupons in both the NON_disinfection_ and UV_disinfection_ reactors became significantly more than that in the NaClO_disinfection_ reactor (*P* < 0.01, paired *t* test). At the end of the 1-year experimental period, the weight loss of the coupons in the NON_disinfection_ and UV_disinfection_ reactors reached 3.53 g ± 0.14 g and 3.57 g ± 0.08 g, which accounted for 19.4% ± 1.1% and 19.7% ± 0.1% of the initial coupon weight, respectively. For the NaClO_disinfection_ reactor, the weight loss was only 2.49 g ± 0.19 g, accounting for 13.5% ± 1.5% of the initial coupon weight.

Polarization curves are frequently used to characterize electrochemical reactions at the metal/biofilm interface and the formation of corrosion and biofilms [15, 36]. In this study, the polarization curves (Additional file 1: Figure S3) of the corroded coupons in the corresponding water were measured to analyze the change in the corrosion current density. Corrosion current density did not exhibit significant discrepancy before the 19th week in the three reactors (*P* > 0.05, paired *t* test), while it was significantly higher in the NON_disinfection_ and UV_disinfection_ reactors than in the NaClO_disinfection_ reactor after the 19th week. The electrochemical results agreed with the weight loss results and confirmed that cast iron coupons suffered much more serious corrosion in the NON_disinfection_ and UV_disinfection_ reactors than did those in the NaClO_disinfection_ reactor in the mid-late experiment period.

#### Morphology and crystal structures of the corrosion scale

**Fig. 2:**
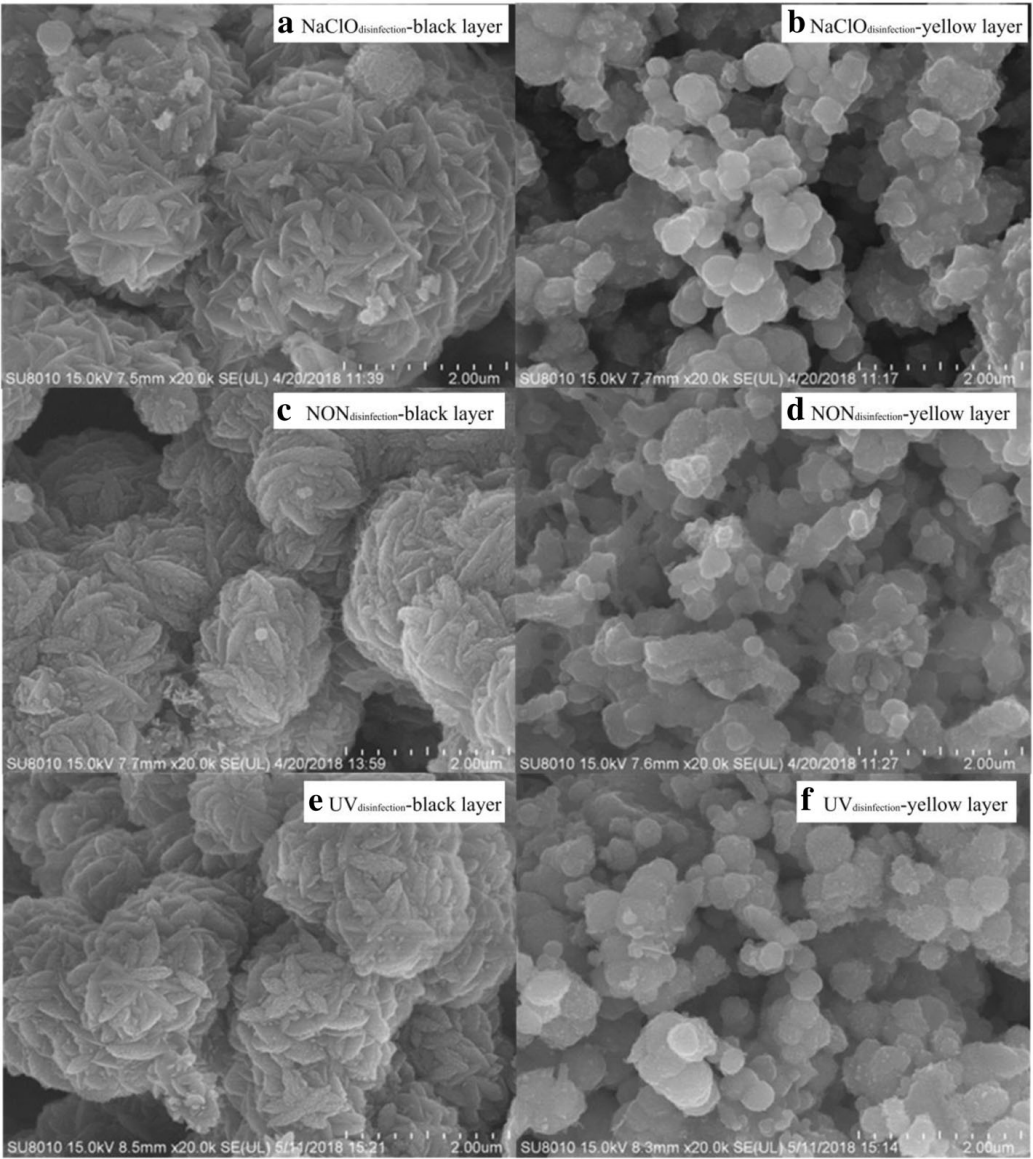
SEM micrograph of the cast iron corrosion scale at the 52nd week, magnification = ×20,000. **a** Black layer in the NaClO_disinfection_ reactor; **b** yellow layer in the NaClO_disinfection_ reactor; **c** black layer in the NON_disinfection_ reactor; **d** yellow layer in the NON_disinfection_ reactor; **e** black layer in the UV_disinfection_ reactor; **f** yellow layer in the UV_disinfection_ reactor

Additional file 1: Figure S2 shows examples of the partitioning of the yellow layer and black layer in the NaClO_disinfection_ and NON_disinfection_ reactors, respectively. Compared to that in the NON_disinfection_ reactor, the corrosion scale of cast iron in the NaClO_disinfection_ reactor was flatter, thinner, and more close-grained. Scanning electron microscopy (SEM) showed that in the corrosion scales of the black layer (Fig. 2a, c, e; Additional file 1: Figure S4a, c, e), the fusiform-shaped nanoparticles were agglomerated into larger spheres with a size of 2 μm. The image of the black layer corrosion scale is very similar to the corrosion scale disinfected using chloramine in drinking groundwater distribution systems [37]. For the yellow layers (Fig. 2b, d, f; Additional file 1: Figure S4b, d, f), the corrosion scales were composed of loose sphere-shaped nanoparticles. Furthermore, the elemental composition of the corrosion scales was detected by SEM and energy-dispersive spectrometer. C, O, Si, Al, and Fe were the predominant elements among the six samples (Additional file 1: Figure S5).

**Fig. 3:**
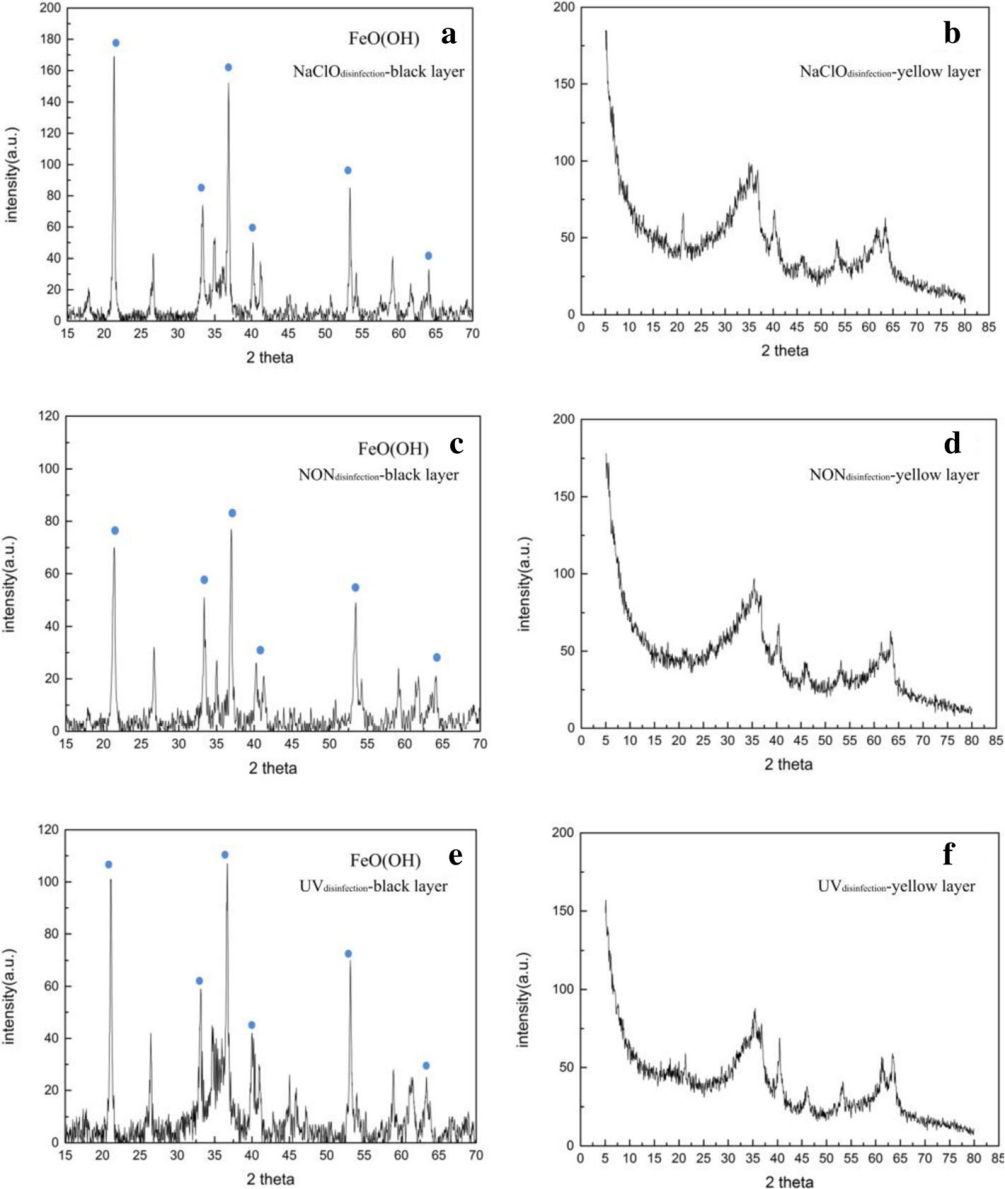
XRD spectrograms of the cast iron corrosion products at the 52nd week. **a** Black layer in the NaClO_disinfection_ reactor; **b** yellow layer in the NaClO_disinfection_ reactor; **c** black layer in the NON_disinfection_ reactor; **d** yellow layer in the NON_disinfection_ reactor; **e** black layer in the UV_disinfection_ reactor; **f** yellow layer in the UV_disinfection_ reactor

An X-ray diffractometer was used to characterize the crystal structure of corrosion scales in the yellow and black layers on cast iron coupons at the 4th week, 34th week, and 52nd week (Additional file 1: Figures S6 and S7, Fig. 3). Goethite (FeOOH) was identified as the predominant crystalline phase in the corrosion scale of the black layers during the entire experimental period regardless of disinfection methods. This was consistent with previous studies, i.e., goethite was the dominant crystalline phase of the cast iron corrosion scale in both drinking water and reclaimed wastewater distribution pipelines [3, 5]. However, in addition to goethite, magnetite, siderite, lepidocrocite, and calcite were also detected in the cast iron corrosion scales, reported by Wang et al. [3] and Zhu et al. [10], but these crystalline phases did not appear in our study. For the yellow layers, corrosion products mainly exhibited amorphous structures during the entire experiment period. It should be noted that cast iron corrosion scales were not divided into yellow layers and black layers for subsequent morphology and crystal structure characterization in previous studies. Pretreatment of the partition of the yellow layer and black layer could provide more specific and accurate information on the morphology and crystal structures for cast iron corrosion scales.

To decipher why the cast iron coupons in the UV_disinfection_ and NON_disinfection_ reactors suffered more serious corrosion than did those in the NaClO_disinfection_ reactor, water quality parameters, microbial quantity, microbial activity, and community composition were comprehensively analyzed. Among all the water quality parameters detected, the concentrations (or values) of TN, TP, TOC, hardness, soluble iron, and pH in the NaClO_disinfection_, UV_disinfection_, and NON_disinfection_ reactors were highly similar (Additional file 1: Table S1). However, the ORP, free chlorine and total chlorine concentrations in the NaClO_disinfection_ reactor were much higher than those in the UV_disinfection_ and NON_disinfection_ reactors due to the addition of NaClO (Additional file 1: Table S1). Oxidation-reduction potential, representing the oxidizing ability of water, was up to 473.2 ± 62.9 mv in the NaClO_disinfection_ reactor, which was much higher than that in the NON_disinfection_ (290.8 ± 87.1 mv) and UV_disinfection_ (282.0 ± 80.4 mv) reactors, respectively. Theoretical Eh (volts) values for Fe^2+^–γ-goethite couples, Fe^2+^–α-goethite couples, Fe^2+^–Fe_3_O_4_ couples, and Fe–Fe^2+^ couples were 88 mv, 274 mv, 314 mv, and 440 mv at circumneutral pH, respectively [38]. These values were all less than the ORP of the NaClO_disinfection_ water. The existence of ClO^−^ can promote the transition from Fe^2+^ to Fe^3+^ [12]. In summary, greater ORP and the existence of ClO^−^ were beneficial for oxidizing Fe^2+^ to Fe^3+^ and Fe–Fe^2+^, which could have caused more severe corrosion. Nevertheless, cast iron coupons in the NaClO_disinfection_ reactor after the 19th week exhibited weaker corrosion than did the coupons placed in the NON_disinfection_ and UV_disinfection_ reactors. Therefore, it seems that water parameters are not the main factors resulting in differences in corrosion behavior. Instead, microbial quantity, microbial activity, or community composition might be responsible for the corrosion difference.

### Microbial quantity and activity under different disinfection conditions

A flow cytometer was used extensively to count the cell numbers due to its high accuracy [27, 28]. The amounts of live, dead, and total bacteria in the biofilm in black and yellow layers over time were measured separately in the present study. Dead bacteria in the yellow layers of the NaClO_disinfection_, NON_disinfection_ and UV_disinfection_ reactors were always at a low quantity (1.33 × 10^5^ ~  1.29 × 10^7^ cells/cm^2^) throughout the entire experimental period regardless of different disinfection method. Interestingly, the quantity of dead bacteria in the black layers of these three reactors was much higher than that in the corresponding yellow layers (Additional file 1: Figure S8a and d). At the beginning of the experiment (i.e., the first 4–7 weeks for the black layer and the first 10–13 weeks for the yellow layer), the live bacteria in the cast iron biofilm in the NON_disinfection_ and UV_disinfection_ reactors maintained a much more rapid growth rate than did those in the NaClO_disinfection_ reactor. During the initial 22 weeks, the quantity of live microbes in the black layer in the NON_disinfection_ and UV_disinfection_ reactors was also significantly higher than that in the NaClO_disinfection_ reactor (*P* < 0.01, paired *t* test). After the 22nd week, the quantity of viable microbes in the black layer in these three reactors reached the same level and maintained a relatively steady state throughout the whole experimental period (Additional file 1: Figure S8b). For the yellow layers, the quantity of live microbes was higher in the NON_disinfection_ and UV_disinfection_ reactors than in the NaClO_disinfection_ reactor for the initial 10 weeks instead of 22 weeks (*P* < 0.05, paired *t* test, Additional file 1: Figure S8e). The variation in the quantity of total microbial cells over time was similar to that of live microbial cells (Additional file 1: Figure S8c and f).

As shown in Additional file 1: Figure S9, the variation in ATP, which represents microbial activity [24, 27, 39], was highly consistent with the variation in the quantity of live bacteria (Additional file 1: Figure S8b, e). This phenomenon was quite reasonable because ATP reflected the microbial activity of the live cells.

### Diversity and microbial community compositions

#### α-Diversity and β-diversity analyses

In total, 5719 OTUs remained after removing OTUs with an abundance of less than 10 sequences for all 120 samples. The OTU diversity index was expressed by the Chao1 index, Shannon index, and Simpson index (Additional file 1: Figure S10). Chao 1 indexes indicated that, compared to the corresponding black layers in three different reactors, yellow layers had significantly higher OTU richness throughout the entire experimental period (*P* < 0.01, paired *t* test, Additional file 1: Figure S10a). Similar to the Chao1 indexes, the Shannon indexes of the yellow layers were always significantly higher (*P* < 0.01, paired *t* test) than those of the corresponding black layers in the UV_disinfection_ and NON_disinfection_ reactors (Additional file 1: Figure S10b). However, for the NaClO_disinfection_ reactor, black layer samples underwent a drastic diversity increase from 1.78 to 4.37, and the Shannon indexes of the black layer samples began to surpass those of the yellow layer samples after the 10th week. In addition, the Simpson index (Additional file 1: Figure S10c) and Evenness index (Additional file 1: Figure S10d) shared the same trends except, for in the black layer in the NaClO_disinfection_ reactor, where these values suffered a drastic increase during the entire experiment.

It should be noted that both Shannon and Simpson indices of the NaClO_disinfection_-B samples, especially the samples collected after the 7–10 weeks, exhibited higher values as compared to those of UV_disinfection_-B and NON_disinfection_-B samples. It is possible that the chlorination pressure exerted on bacteria in the NaClO_disinfection_-B samples decreased with the increase of corrosion layer thickness because of less contact between bacteria and hypochlorite. This suggested that the NaClO_disinfection_-B samples collected after the 7–10 weeks were under an intermediate pressure level caused by the attenuate chlorination. According to the classical intermediate disturbance hypothesis theory, diversity might reach maximum at intermediate levels of disturbance or pressure [40]. In the present study, the attenuate NaClO disinfection for the NaClO_disinfection_-B samples collected after the 7–10 weeks may act as intermediate disturbance.

A two-dimensional PCoA plot showed the bacterial community differences among the samples in the yellow layers and black layers under different disinfection methods. (Additional file 1: Figure S11). Samples of the black layer were distinctly separated from those of the yellow layer regardless of the disinfection method. One possible reason could be related to the oxygen availability, that is, the yellow layer belongs to the aerobic environment while the black layer might be anaerobic. In addition, compared to the NaClO_disinfection_ reactor, the NON_disinfection_ and UV_disinfection_ reactors had much more similar microbial compositions and variation tendencies over time for the same layer type. The dynamic change in the microbial community also followed a regular tendency and will be discussed in detail in subsection “Community temporal trajectories and identification of differential bacterial genera during biofilm development.”

#### Characterization of microbial community compositions

As shown in Additional file 1: Figure S12, *Proteobacteria* was the most abundant phylum in all the samples collected from both the yellow layers and black layers, accounting for 53.8 ~ 94.2% of the total bacterial community. This is consistent with the analytical results of the bacterial community in the cast iron corrosion scale of RWDS [8] and DWDS [5], in which *Proteobacteria* accounted for 56.7% and 64.0% on average. Another interesting phenomenon is that the relative abundance of *Proteobacteria* in black layers was significantly higher than that in the corresponding yellow layers under different disinfection conditions. In contrast to *Proteobacteria*, *Acidobacteria* is a dominant phylum without significant differences among NaClO_disinfection_ (black layer 4.55% ± 1.80%, yellow layer 5.16% ± 2.17%), NON_disinfection_ (black layer 4.84% ± 2.08%, yellow layer 5.98% ± 1.46%), and UV_disinfection_ (black layer 4.59% ± 1.96%, yellow layer 5.67% ± 1.21%) reactors (*P* > 0.05, paired *t* test). The relative abundance of *Bacteroidetes* in the NaClO_disinfection_ reactor (black layer 5.87% ± 2.26%, yellow layer 8.22% ± 4.92%) was significantly (*P* < 0.01, paired *t* test) higher than that in the NON_disinfection_ (black layer 3.27% ± 2.11%, yellow layer 4.60% ± 2.22%) and UV_disinfection_ (black layer 3.27% ± 2.02%, yellow layer 4.65% ± 2.64%) reactors. Additionally, the relative abundance of *Bacteroidetes* decreased with time, especially in the NaClO_disinfection_ reactor. *Bacteroidetes* are known to produce EPS [41], which can act as a protective mechanism for bacteria in an adverse or stressful environment and contribute to the formation of biofilms. This should be the possible reason for the abundant *Bacteroidetes* in the NaClO_disinfection_ reactor due to the existence of chlorination oxidation stress. Nevertheless, according to the two-month preliminary experiment, we found that it was impracticable to measure the EPS content in the present study because the amount of EPS was not sufficient for the subsequent measurement of protein and polysaccharide, and thus, the contribution of EPS for biofilm formation is difficult to determine. *Nitrospirae* was much more abundant in the NON_disinfection_ yellow layer (5.75% ± 3.78%) and UV_disinfection_ yellow layer (5.93% ± 3.57%) than in the corresponding black layers (0.49% ± 0.31%; 0.58% ± 0.38%), which may be related to its aerobic property. Moreover, it seems that *Nitrospirae* should be very sensitive to chlorination disinfection because its relative abundance in the NaClO_disinfection_ yellow layer was much lower than that in the yellow layers of the NON_disinfection_ and UV_disinfection_ reactors. Similar to *Nitrospirae*, *Actinobacteria* was also much more abundant in the NON_disinfection_ yellow layer (2.15% ± 1.04%) and UV_disinfection_ yellow layer (2.32% ± 1.44%) than in the corresponding black layers (0.76% ± 0.38%; 0.78% ± 0.51%).

At the genus level, Additional file 1: Figure S13 shows the relative abundance distribution of the top 50 genera in all black and yellow samples. *Azospira*, *Sediminibacterium*, *Geothrix*, and *Nitrospira* presented notable differences between the yellow and black layers under different disinfection methods. These potential functional bacteria responsible for corrosion will be discussed in detail in subsection “Identification of crucial genera responsible for promoting cast iron corrosion and establishment of a cycle model for Fe, N and S metabolism.”

It should be noted that, the diversity in NaClO_disinfection_-black layer illustrated in Additional file 1: Figure S12 seems like lower than that in the yellow layer, which is contrast to the Shannon and Simpson indices trend (Additional file 1: Figure S10). The apparent contradiction is explicable and reasonable because Shannon and Simpson indices were calculated based on OTUs (identity threshold of 97%) results, while Additional file 1: Figure S12 was presented at phylum level.

#### Abundant and persistent bacteria during the biofilm development process

**Fig. 4:**
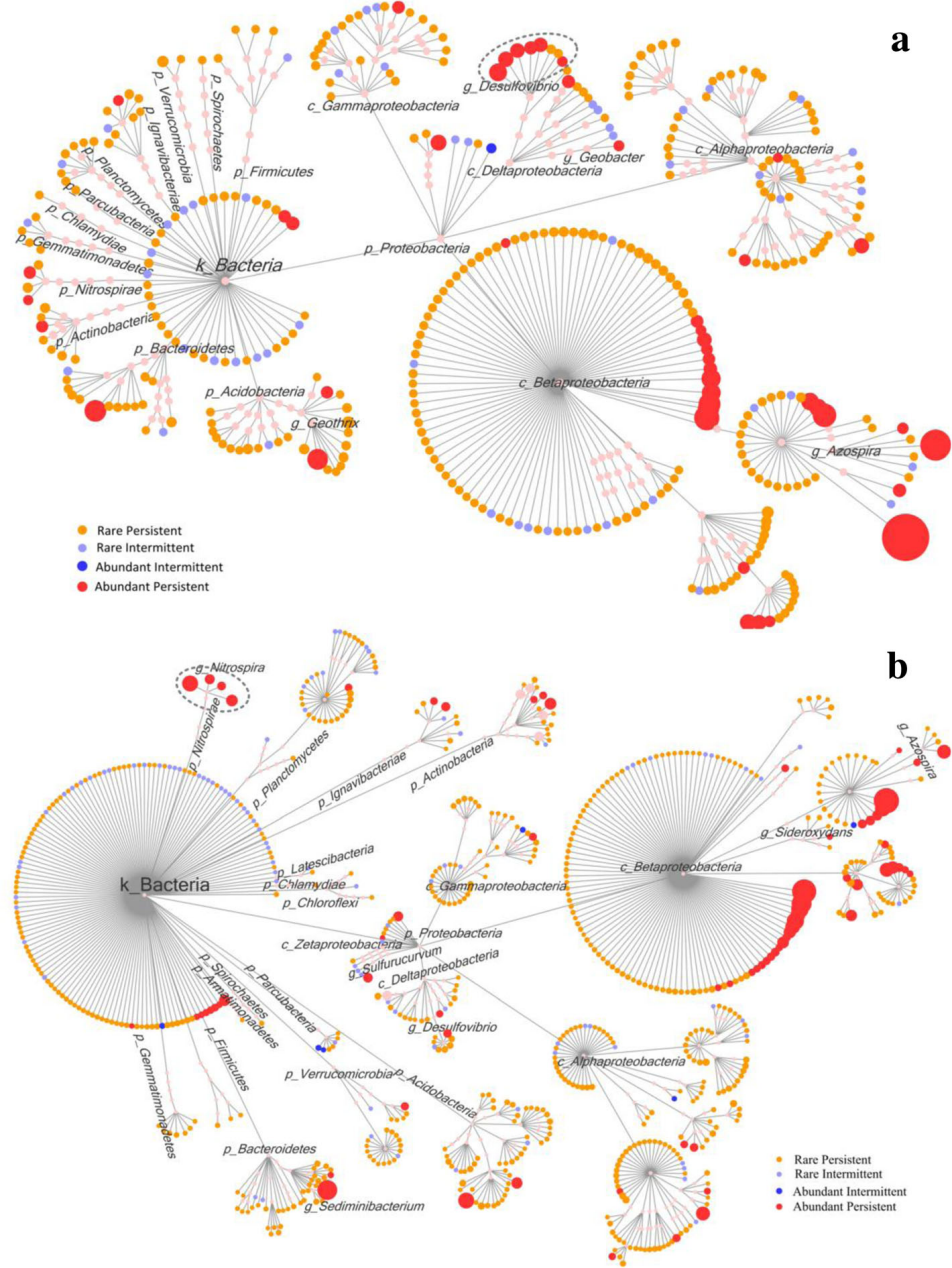
Taxonomic dendrograms of the bacterial community detected over a 1-year period in the **a** black layer and **b** yellow layer of three reactors. Different taxonomic branches are labeled according to phylum, except *Proteobacteria*, which was labeled by class. The edges represent the taxonomic path from the root bacteria down to the OTU level (similarity cutoff: 97%). OTUs were located at the lowest possible assignment level, and the node sizes indicated their relative abundance. The nodes are colored according to both their abundance and frequency of occurrence. Red nodes: abundant-persistent (AP) OTUs; blue nodes: abundant-intermediate (AI) OTUs; orange nodes: rare-persistent (RP) OTUs; violet nodes: rare-intermediate (RI) OTUs. The definition of OTUs types was described in subsection “Abundant and persistent bacteria during the biofilm development process”

Figure 4 shows the taxonomic identity of all OTUs with an abundance sum from 60 samples of more than 0.5% in the black and yellow layers, respectively. We followed the taxonomic composition of bacterial populations from 60 samples retrieved over a 1-year period. To provide a more detailed characterization of the identified OTUs, we classified them into abundant or rare and into persistent, intermediate, and transient types, assuming that abundant and persistent bacterial groups play the most significant roles in biological processes related to corrosion. Abundant OTUs were defined as those that contributed ≥ 1% of the total abundance at least once in all the sampling times, while rare OTUs contributed < 1% of the total abundance in all samples. Persistent OTUs were defined as those detected in ≥ 75% of the samples; intermediate OTUs were detected in 25–75% of the samples, and transient OTUs were detected in < 25% of the samples [42, 43]. We can obtain six types of OTUs: abundant-persistent (AP), rare-persistent (RP), abundant-intermediate (AI), rare-intermediate (RI), abundant-transient (AT), and rare-transient (RT) OTUs. In total, 390 OTUs (accounting for 95.5% ± 2.1% of the total bacterial abundance) for the black layer (Fig. 4a) and 742 OTUs (accounting for 92.4% ± 3.8% of the total bacterial abundance) for the yellow layer (Fig. 4b) were selected for the taxonomic dendrogram analysis.

Among the 390 OTUs in the black layer, 45 AP-type OTUs accounted for 85.0% ± 5.6% of the total bacterial abundance. One OTU, 52 OTUs, and 292 OTUs were classified as AI, RI, and RP, respectively. These 45 AP-type OTUs belonged mainly to the six phyla of *Acidobacteria*, *Actinobacteria*, *Bacteroidetes*, *Nitrospirae*, *Ignavibacteriae*, and *Proteobacteria.* Among the 742 OTUs screened in the yellow layer, 74 AP-type OTUs belonging to eight phyla accounted for 72.1% ± 11.0% of the total bacterial abundance. These AP-type OTUs were classified to 8 phyla, including the 6 phyla mentioned above and *Planctomycetes* and *Verrucomicrobia*. Except for AP-type OTUs, 6 OTUs, 93 OTUs, and 569 OTUs were classified into AI, RI, and RP types, respectively. It should be noted that there were no AT-type or RT-type OTUs in either the black or yellow layer samples.

Among the four classes of *Proteobacteria*, branches of *β-Proteobacteria* had 51% and 51.4% of AP-type OTUs, irrespective of being from black or yellow layer samples (red dots in Fig. 4). It is worth mentioning that in six out of eight OTUs belonging to *Desulfovibrio*, one type of well-known SRB genus [44] was AP-type OTU in the black layer. In the yellow layer, four OTUs derived from *Nitrospira* responsible for nitrite oxidizing were all AP-type OTUs. Other functional genera will be discussed in detail in subsection “Identification of crucial genera responsible for promoting cast iron corrosion and establishment of a cycle model for Fe, N and S metabolism.”

### Community temporal trajectories and identification of differential bacterial genera during biofilm development

#### Community temporal trajectories and biofilm development stage divide

**Fig. 5:**
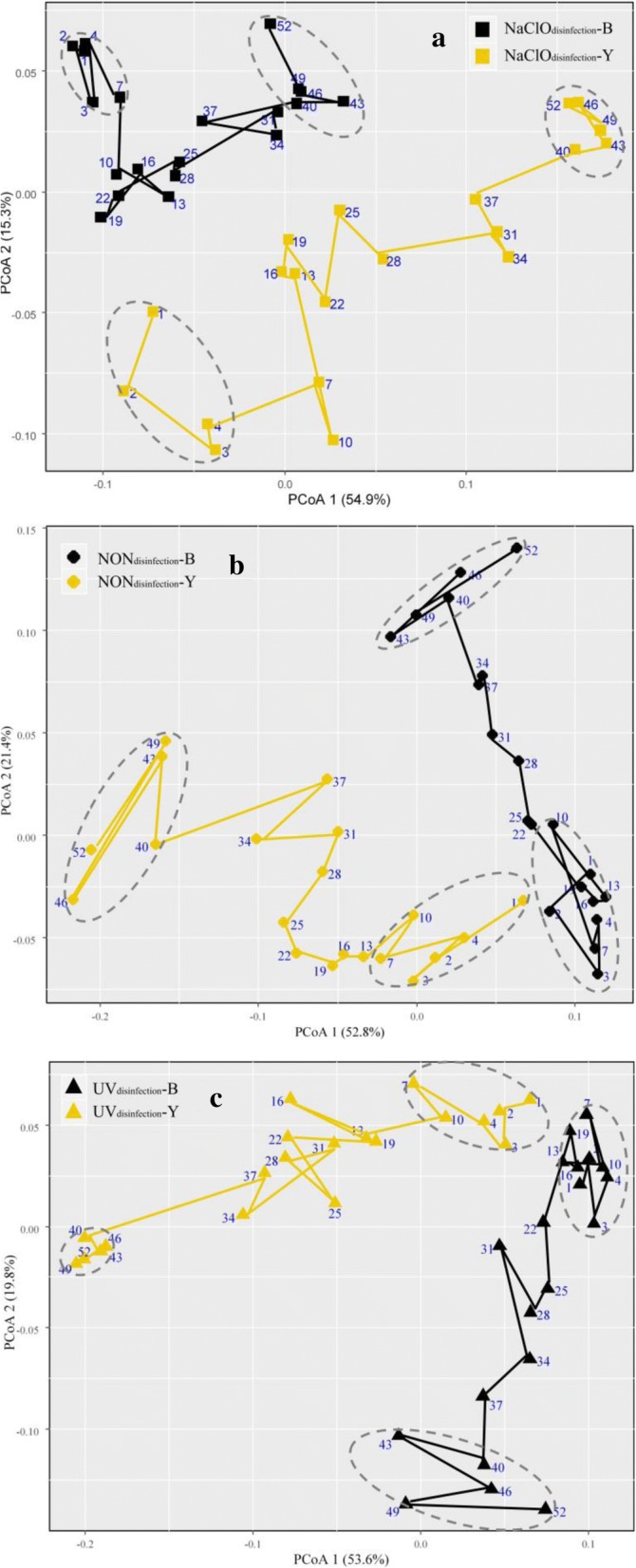
Temporal trajectories in the community composition of the yellow and black layers are presented in the ordination space of principal coordinate analysis (PCoA) based on weighted UniFrac distance for the **a** NaClO_disinfection_ reactor, **b** NON_disinfection_ reactor, and **c** UV_disinfection_ reactor. Trajectories were presented by lines that sequentially connect sampling points. Circles highlighted initial attachment stage I and terminal stable stage III. Other plots were classified as mid-term development stage II

To explore the dynamic trend of microbial composition in both black and yellow layers under three disinfection conditions over a 1-year period, three trajectory graphs were presented in the ordination space of PCoA based on weighted UniFrac distance [35]. Trajectories were presented by lines sequentially connecting sampling points. Pairwise comparisons of community composition shifting through time indicated that bacterial communities exhibited similar trajectories in both black and yellow layers under the three disinfection conditions. In the early stage of the experiment, samples fluctuated and moved slightly along with the two principal coordinate axes, then shifted drastically in the mid-term stage, and finally became relatively stable with negligible fluctuation in the terminal stage. According to the three trajectory graphs shown in Fig. 5, biofilm development can be divided into three stages: initial start-up stage (stage I), mid-term development stage (stage II), and terminal stable stage (stage III). Stage III suggested that the bacterial community compositions reached a final steady phase during the entire experimental period. To conduct downstream comparison analysis, stages I and III are highlighted by gray-dotted ellipses in Fig. 5.

#### Identification of differential bacterial genera during biofilm development

**Fig. 6:**
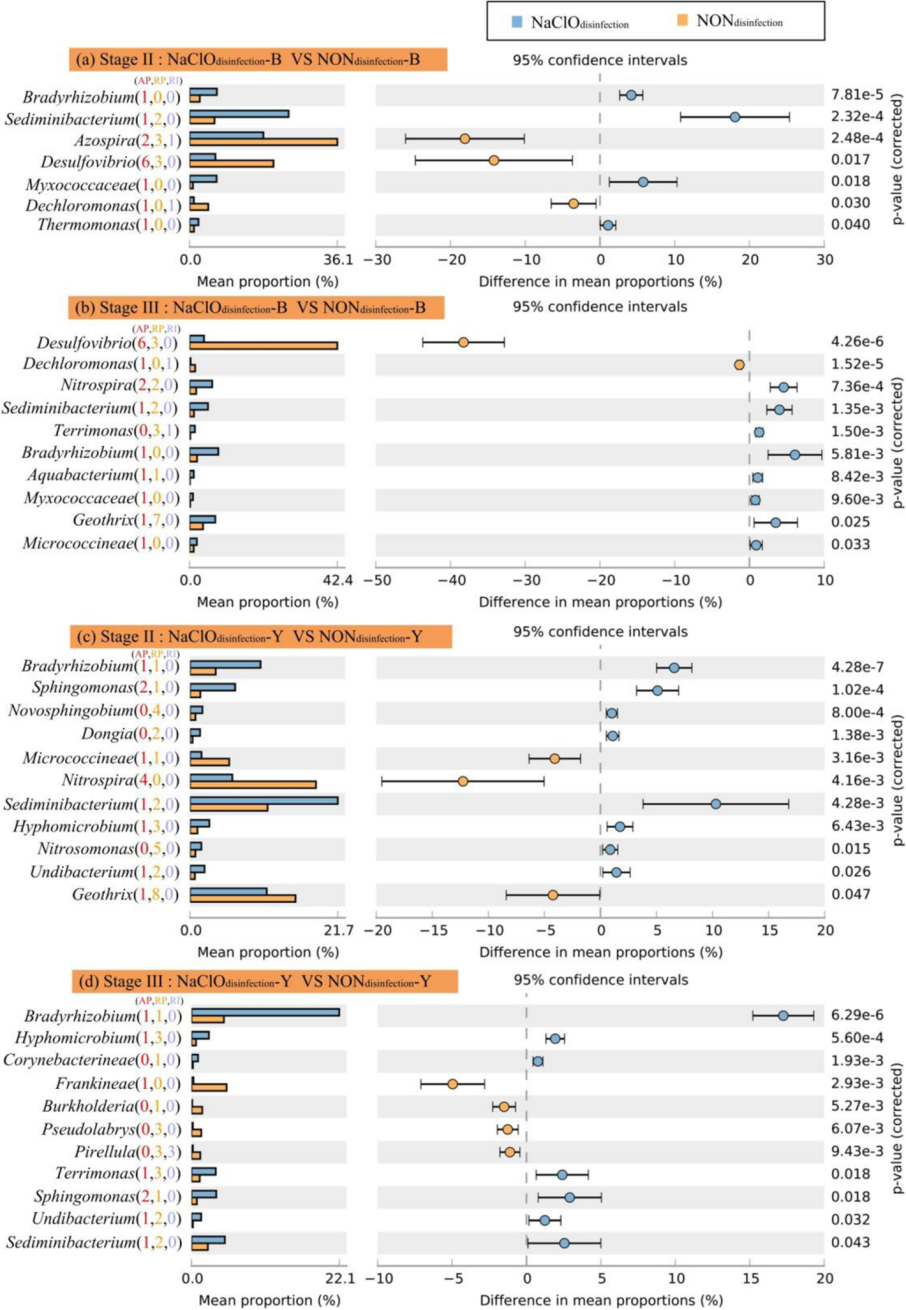
Extended error bar plots showing the abundance of genera differing significantly between the NaClO_disinfection_ and NON_disinfection_ reactors with an effect size of 0.75. **a** Genera in the black layer of stage II; **b** genera in the black layer of stage III; **c** genera in the yellow layer of stage II; **d** genera in the yellow layer of stage III. The numbers in the parentheses represent the amounts of OTUs belonging to the genus correspondingly to Fig. 4. The red numbers represent the AP-type OTUs; the orange numbers represent the RP-type OTUs; and the light purple numbers represent the RI-type OTUs

STAMP (statistical analysis of taxonomic and functional profiles) is a powerful software tool to test for differences between two groups using mean proportion effect size measures along with Welch’s confidence intervals [45]. As shown in Fig. 6 and Additional file 1: Figure S14, a two-group Welch *t* test was conducted among different disinfection reactors in both the black and yellow layers, respectively, based on the three stages with an effect size ≥ 0.75 and *P* value < 0.05 [45]. Considering the difference in weight loss starting from the 19th week in the three reactors (Fig. 1) and the biofilm development stage divide (Fig. 5), the bacterial composition differences of stages II and III between the NaClO_disinfection_ reactor and the other two reactors were concerning.

During stage II, seven genera, i.e., *Bradyrhizobium*, *Azospira*, *Sediminibacterium*, *Myxococcaceae*, *Desulfovibrio*, *Thermomonas*, and *Dechloromonas*, differed significantly between the black layers in the NaClO_disinfection_ and NON_disinfection_ reactors (Fig. 6a). This was very similar to the comparison between the black layers in the NaClO_disinfection_ and UV_disinfection_ reactors, apart from the addition of *Micrococcineae* (Additional file 1: Figure S14a). For the yellow layer, 11 genera, including *Bradyrhizobium*, *Sphingomonas*, *Novosphingobium*, *Dongia*, *Micrococcineae*, *Nitrospira*, *Sediminibacterium*, *Hyphomicrobium*, *Nitrosomonas*, *Undibacterium*, and *Geothrix*, manifested significant differences between the NaClO_disinfection_ and NON_disinfection_ reactors (Fig. 6c). In addition to the above genera, *Opitutus* and *Aquabacterium* also exhibited significant differences between the NON_disinfection_ and UV_disinfection_ reactors (Additional file 1: Figure S14c). With an effect size ≥ 0.75 recommended by Parks et al. [45], no genus significantly differed between the NON_disinfection_ and UV_disinfection_ reactors, regardless of being from the black or yellow layers.

During stage III, ten genera, including *Desulfovibrio*, *Dechloromonas*, *Nitrospira*, *Sediminibacterium*, *Terrimonas*, *Bradyrhizobium*, *Aquabacterium*, *Myxococcaceae*, *Geothrix*, and *Micrococcineae*, differed significantly in the black layers between the NaClO_disinfection_ and NON_disinfection_ reactors (Fig. 6b). Except for *Micrococcineae*, the other nine genera differed significantly between the black layers in the NaClO_disinfection_ and UV_disinfection_ reactors (Additional file 1: Figure S14b). With regard to the comparison of the yellow layers in the NaClO_disinfection_ and NON_disinfection_ reactors (Fig. 6d), 11 genera, i.e., *Bradyrhizobium*, *Hyphomicrobium*, *Corynebacterineae*, *Frankineae*, *Burkholderia*, *Pseudolabrys*, *Pirellula*, *Terrimonas*, *Sphingomonas*, *Undibacterium*, and *Sediminibacterium*, showed distinct differences. However, 13 genera, including *Bradyrhizobium*, *Nitrospira*, *Pirellula*, *Hyphomicrobium*, *Corynebacterineae*, *Gaiellaceae*, *Sediminibacterium*, *Sphingomonas*, *Melioribacter*, *Burkholderia*, *Frankineae*, *Terrimonas*, and *Undibacterium*, displayed statistically significant differences between the NaClO_disinfection_ and UV_disinfection_ reactors (Additional file 1: Figure S14d). Two group tests between the NON_disinfection_ and UV_disinfection_ reactors indicated that only one genus displayed a significant difference, i.e., *Azospira* corresponding to the black layer and *Frankineae* corresponding to the yellow layer.

### Identification of crucial genera responsible for promoting cast iron corrosion and establishment of a cycle model for Fe, N and S metabolism

**Fig. 7:**
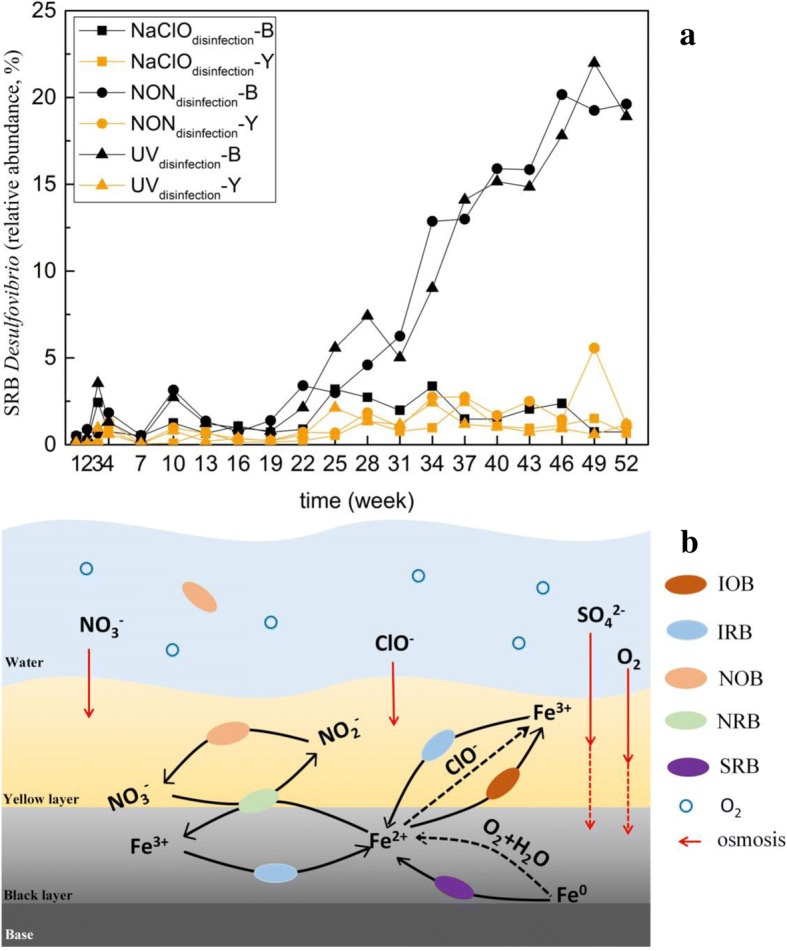
**a** The abundance variation in *Desulfovibrio* during a 1-year period. **b** Model of the redox transition between NO_3_^−^ and NO_2_^−^, Fe, Fe^2+^, and Fe^3+^. The electrochemical corrosion process is represented by black dotted lines, and the microbial-induced corrosion process is represented by black solid lines

In total, 26 genera were found to have significant differences between the NaClO_disinfection_ reactor and the other two reactors (Fig. 6 and Additional file 1: Figure S14). According to the literature review, 12 out of 26 genera were potential functional species playing roles in the cast iron corrosion process in RWDS. These 12 genera mainly included four nitrate-dependent IOBs: *Aquabacterium* [46, 47], *Sediminibacterium* [48], *Azospira* [49], and *Geobacter* [50]; one IRB: *Geothrix* [51, 52]; five nitrate-reducing bacteria (NRBs): *Thermomonas* [53], *Rhodoferax* [53], *Sulfuritalea* [53], *Dechloromonas* [53], and *Hyphomicrobium* [54]; one nitrite-oxidizing bacteria (NOB): *Nitrospira* [55]; and one SRB: *Desulfovibrio* [56]*.* To obtain a clearer picture of the variability in the functional genera in different layers and reactor systems, relative abundance variation with time was portrayed for IOBs, IRBs, NOBs, NRBs (Additional file 1: Figure S15), and SRBs (Fig. 7), respectively.

#### IOBs and IRBs

The total relative abundance of IOBs, i.e., *Azospira*, *Aquabacterium*, *Geobacter*, and *Sediminibacterium*, increased distinctly in all black layers over time and decreased in yellow layers (Additional file 1: Figure S15a). *Azospira* is able to oxidize iron (II) using nitrate as an electron acceptor instead of oxygen [49]. The decrease in the total relative abundance of IOBs in the yellow layer was caused by *Sediminibacterium*, which suffered a gradual reduction in all yellow layer samples regardless of disinfection method (Additional file 1: Figure S15e). *Sediminibacterium* is always isolated from sediment and activated sludge [57]. It tends to grow under aggregation conditions to protect bacteria against oxidative stress [58], and this aggregation property may be responsible for its high relative abundance in the initial stage due to contributing to the formation of biofilms. The decreased relative abundance of *Sediminibacterium* over time may be caused by an increasingly complex microbial community structure, which leads to fiercer interspecific competition. *Geobacter* was found to be the first microorganism with the ability to oxidize organic compounds and metals, including iron. One *Geobacter* species, *Geobacter metallireducens*, has the ability to oxidize Fe(II) using nitrate as electron acceptor and generate ammonium [59], which demonstrated that nitrite (NO_2_^−^) and nitrogen gas (N_2_) were not the sole end products of nitrate reduction. In the present study, the relative abundance of *Geobacter* remained less than 0.2% before the 25th week and then increased unstably (Additional file 1: Figure S15f). *Geothrix*, as an IRB [51, 52], was widespread in any layer and fluctuated slightly during the entire experimental period (Additional file 1: Figure S15b).

#### NOBs and NRBs

Nitrite-oxidizing bacteria, such as *Nitrospira*, carried out two obvious ascend phases in the yellow layers of the NON_disinfection_ and UV_disinfection_ reactors (Additional file 1: Figure S15c), while the relative abundance of NOBs in the yellow layer of the NaClO_disinfection_ reactor remained relatively stable before the 31st week and then increased continuously. Nitrate-reducing bacteria spread across multiple prokaryotic phyla with diverse physiologies [60]. Five genera belonging to NRBs with significant relative abundance differences were filtered out by STAMP analysis. The total relative abundance did not significantly differ in the three reactors regardless of the black or yellow layers (Additional file 1: Figure S15d).

#### SRBs

The relative abundance of SRBs, i.e., *Desulfovibrio*, increased from the 22nd week in the black layer of the NON_disinfection_ and UV_disinfection_ reactors (Fig. 7a), which coincided with the variation in weight loss results (Fig. 1). Although both electrochemical corrosion and microbiological-induced corrosion existed in the NaClO_disinfection_ and NON_disinfection_ reactors, NaClO_disinfection_ could have influenced the overall microbial community composition to the extent that the influence of microbiological-induced corrosion was reduced. Additionally, due to the higher oxidation-reduction potential in the NaClO_disinfection_ reactors, the cast iron in NaClO_disinfection_ reactors should suffer more severe electrochemical corrosion than that in NON_disinfection_ reactor. Combining these two factors, the cast iron in NON_disinfection_ reactor exhibited more weight losses after 19th week. Therefore, via ignoring electrochemical corrosion difference between NON_disinfection_ reactor and NaClO_disinfection_ reactor, the least contribution of the bacterial community to cast iron corrosion in NON_disinfection_ reactors can be estimated according to Eq. (1). From the 22nd to 52nd weeks, bacterial community-induced corrosion accounted for at least 30.5% ± 9.7% of the total weight loss in the NON_disinfection_ reactor. Sulfate-reducing bacteria can notably influence iron (Fe^0^) corrosion in anaerobic environments, and the mechanism is usually explained by the corrosiveness of formed H_2_S and the scavenge of “cathodic” H_2_ from the chemical reaction of Fe^0^ with H_2_O [61]. Among SRBs, *Desulfovibrio* species are conventionally regarded as the main culprits of anaerobic corrosion because of their capability to consume hydrogen effectively [62]. It has been verified that *Desulfovibrio* indeed have derepressed hydrogenase to consume cathodic hydrogen on the metal surface and thus accelerate the dissolution of Fe^2+^ from the anode to ensure a ready supply of Fe^2+^ for the cells’ iron proteins under stressful conditions [56]. In the present study, the concentration of SO_4_^2−^ in the effluent (not shown) was lower in the NON_disinfection_ and UV_disinfection_ reactors than in the NaClO_disinfection_ reactor (*P* < 0.01, paired *t* test), which can be proof of SRBs’ higher activity in the NON_disinfection_ and UV_disinfection_ reactors. Therefore, the existence of *Desulfovibrio* was suggested to accelerate the transfer of Fe^0^ to Fe^2+^, speed up the loss of weight, and result in more serious corrosion.

1 $$ \mathrm{Contribution}\ \mathrm{of}\ \mathrm{bacteria}-\mathrm{induced}\ \mathrm{corrosion}=\frac{\frac{{\mathrm{W}\mathrm{L}}_{\mathrm{NON}}}{{\mathrm{W}}_{\mathrm{O}}}-\frac{{\mathrm{W}\mathrm{L}}_{\mathrm{NaClO}}}{{\mathrm{W}}_{\mathrm{O}}}}{\frac{{\mathrm{W}\mathrm{L}}_{\mathrm{NON}}}{{\mathrm{W}}_{\mathrm{O}}}}\times 100\% $$

Where WL_NON_ represents the weight loss of the cast iron coupon in the NON_disinfection_ reactor, WL_NaClO_ represents the weight loss of the cast iron coupon in the NaClO_disinfection_ reactor and W_O_ represents the original weight of the cast iron coupon.

#### Cycle model for Fe, N, and S metabolism

Nitrate-dependent IRBs, such as *Geobacter*, *Sediminibacterium*, and *Azospira*, can oxidize Fe^2+^ to Fe^3+^ accompanied by the reduction of nitrate to nitrite [38, 49], and then, the nitrite is oxidized by NOBs to generate nitrate. Iron-reducing bacteria, such as *Geothrix*, can reduce Fe^3+^ to Fe^2+^. Iron-oxidizing bacteria/NRB, IOBs/NRBs, IRBs, and NOBs constituted a cycle model for Fe, N, and S metabolism (Fig. 7b) to drive the transition between NO_3_^−^ and NO_2_^–^, Fe^2+^, and Fe^3+^. The redox transition between Fe^2+^ and Fe^3+^ did not contribute to the weight loss, while only the dissolution of Fe to Fe^2+^ increased the weight loss. Oxygen can be used as an electron acceptor to oxidize Fe to Fe^2+^ by galvanic effect, which was the primary pathway to dissolve Fe in the initial experimental stage in all three reactors. However, the ability of oxygen penetration was limited by the increased corrosion layer thickness. Our previous study found that oxygen was blocked effectively, and anaerobic conditions could be created when the corrosion layer thickness of cast iron was greater than ~ 8 mm [8]. When the corrosive layer blocked oxygen penetration, it also created an appropriate environment for anaerobic bacteria at the same time. Sulfate-reducing bacteria are anaerobic [62], and their survival depends on the corrosion layer, which prevents oxygen from entering. The corrosion layer was thinner in the NaClO_disinfection_ reactor (Additional file 1: Figure S2a) than in the NON_disinfection_ reactor (Additional file 1: Figure S2b). The thinner corrosion layer was insufficient to inhibit oxygen definitely; thus, only a few *Desulfovibrio* could survive in the black layer, which resulted in slight or negligible microbiology-induced corrosion. NaClO could oxidize Fe^2+^ to Fe^3+^ in the NaClO_disinfection_ reactor. Even if the relative abundance of IOBs in the NaClO_disinfection_ reactor was lower than that in the NON_disinfection_ and UV_disinfection_ reactors in the initial stage, the reaction of Fe^2+^ to Fe^3+^ also existed in the corrosion scale of the NaClO_disinfection_ reactor (Fig. 3 and Additional file 1: Figure S6). Overall, although the disinfection of NaClO theoretically enhanced the electrochemical corrosion of cast iron, it inhibited the microbiology-induced corrosion simultaneously by influencing the thickness of the corrosion layer and different microbiology compositions.

Nevertheless, this circle model mainly takes into account some bacteria with known functions related to Fe, N, and S metabolism and possessing significant relative abundance differences between the NaClO_disinfection_ and NON_disinfection_ reactors. In addition, a large portion of the effective bacterial sequences in this study could not be assigned to the genus level. Overall, it is not sufficient to construct the above conceptual model and predicate the effect of SRBs based on 16S rRNA gene annotation results alone. If a more accurate mechanism hypothesis is established, it is suggested to confirm the gene abundance variation and expression levels involving Fe, N, and S metabolisms using metagenomics and metatranscriptomics approaches. Moreover, the protection function of EPS which might play a possible role in inhibiting corrosion should also be considered in the future study.

## Conclusions

The long-term effect of disinfection processes on the corrosion behaviors of cast iron in RWDS and the related hidden mechanisms were deciphered in the present study. The cast iron coupons in the NON_disinfection_ and UV_disinfection_ reactors suffered more serious corrosion than did those in the NaClO_disinfection_ reactor, while there was no significant difference in corrosion behaviors between the NON_disinfection_ and UV_disinfection_ reactors. Bacterial community composition was considered the principal factor resulting in the different corrosion behaviors, and the corrosion induced by the bacterial community accounted for 30.5% ± 9.7% of the total weight loss in the NON_disinfection_ reactor. The partition of the yellow layer and black layer of the cast iron corrosion scales provided more specific and accurate information on the morphology, crystal structures, and bacterial community compositions for corrosion scales. *Proteobacteria* was the most abundant phylum, accounting for 53.8 ~ 94.2% of the total bacterial community in the corrosion scale samples, followed by *Acidobacteria*, *Bacteroidetes*, and *Nitrospirae*. Core bacterial community, i.e., AP-type OTUs, existed during the 1-year dynamic period, with relative abundance accounting for 85.0% ± 5.6% and 72.1% ± 11.0% of the total bacterial relative abundance in the black and yellow layers, respectively. Twelve functional genera, including four IOBs, one IRB, five NRBs, one NOB, and one SRB, were selected to establish a cycle model for Fe, N, and S metabolism. Iron-oxidizing bacteria, NRBs, IRBs, and NOBs drove the transition between NO_3_^−^ and NO_2_^–^, Fe^2+^, and Fe^3+^. Oxygen acted as an electron acceptor to oxidize Fe to Fe^2+^ by galvanic effect, which was the primary pathway to dissolve Fe in all three reactors. Except for the above electrochemical corrosion process, *Desulfovibrio* was considered to accelerate the transfer of Fe^0^ to Fe^2+^ and thus result in more serious corrosion in the NON_disinfection_ and UV_disinfection_ reactors.

## Additional file

Additional file 1: The additional file accompanying this article contains **Figures S1–S15** and **Tables S1–S2**. (DOCX 23899 kb)

## Abbreviations

AI: Abundant-intermediate
AP: Abundant-persistent
AT: Abundant-transient
TP: Adenosine triphosphate
DWDS: Drinking water distribution system
EPS: Extracellular polymeric substance
IOBs: Iron-oxidizing bacteria
IRBs: Iron-reducing bacteria
NaClO_disinfection_: Sodium hypochlorite treated
NOBs: Nitrite-oxidizing bacteria
NON_disinfection_: Without disinfection treatment
NRBs: Nitrate-reducing bacteria
RI: Rare-intermediate
RP: Rare-persistent
RT: Rare-transient
RWDS: Reclaimed wastewater distribution system
SOBs: Sulfur-oxidizing bacteria
SRBs: Sulfate-reducing bacteria
UV_disinfection_: UV treated
