# Supplementary material for: The bacterial community significantly promotes cast iron corrosion in reclaimed wastewater distribution systems
Source: Microbiome. 2018 Dec 13;6:222. doi: 10.1186/s40168-018-0610-5 (PMC6292113; doi:10.1186/s40168-018-0610-5)
Supplement: Supplementary file 1 — The additional file accompanying this article contains Figures S1–S15 and Tables S1–S2. (DOCX 23899 kb) [file 40168_2018_610_MOESM1_ESM.docx]

**The bacterial community significantly promotes cast iron corrosion in reclaimed wastewater distribution systems**

Guijuan Zhang^1,2^, Bing Li^1,2^*, Jie Liu^1,2^, Mingqiang Luan^1,2^, Long Yue^1,2^, Xiaotao Jiang^3^, Ke Yu^4^, Yuntao Guan^1,2^*

^∗^Corresponding author: Bing Li, bingli@sz.tsinghua.edu.cn;

Yuntao Guan, guanyt@sz.tsinghua.edu.cn

^1^ Guangdong Provincial Engineering Research Center for Urban Water Recycling and Environmental Safety, Graduate School at Shenzhen, Tsinghua University, China

^2^ State Environmental Protection Key Laboratory of Microorganism Application and Risk Control, School of Environment, Tsinghua University, Beijing, China

^3^ Microbiome Research Centre, St George and Sutherland Clinical School, University of New South Wales, Australia

^4^ School of Environment and Energy, Graduate School, Peking University, Shenzhen, China

**Supplementary Information**

**Text S1:** **Detection method summary of water quality**

**Text S2: Evaluation of the effect of sonication pretreatment on the bacterial cell viability and ATP measurement**

**List of tables**

**Table S1** Water quality parameters of NaClO_disinfection_ reclaimed wastewater, NON_disinfection_ reclaimed wastewater and UV_disinfection_ reclaimed wastewater.

**Table S2** Sampling time points

**List of figures**

**Figure S1** (a) Schematic experimental setup used for laboratory-scale study. The effective size of each reactor is 90 cm×30 cm×10 cm (length×width×depth). There are three lanes in each reactor with the effective size of 90 cm×30 cm×10 cm (length×width×depth).The arrow represents the water flow direction and the flow rate was 0.2 m/s. (b) The actual picture showing one lane (90 cm×30 cm×10 cm) of the NaClO_disinfection_ reactor.

**Figure S2** Surface (yellow) layer and inner (black) layer were defined according to its color. (a) Cast iron coupon in NaClO_disinfection_ reactor at 52nd week. The thickness of the yellow layer and black layer is approximately 2.0~2.5 mm and 0.5~1 mm, respectively; (b) Cast iron coupon in NON_disinfection_ reactor at 52nd week. The thickness of the yellow layer and black layer is approximately 7.5~9.5 mm and 2.0~3.0 mm, respectively. The thickness of the underlying cast iron bases in the NaClO_disinfection_ reactor and the NON_disinfection_ reactor were 1.71-1.75 mm and 1.60-1.63 mm, respectively.

**Figure S3** (a) The corrosion current of cast iron coupons in NaClO _disinfection_, UV _disinfection_ and NON _disinfection_ reactors over one year; Polarization curves of cast iron coupons in (b) NaClO_disinfection_ reactor, (c) NON _disinfection_ reactor and (d) UV _disinfection_ reactor. Data points indicates n=1, curves indicate n=1.

**Figure S4** SEM micrograph of the cast iron corrosion scale at 52nd week. (a) black layer in NaClO_disinfection_ reactor; (b) yellow layer in NaClO_disinfection_ reactor; (c) black layer in NON_disinfection_ reactor; (d) yellow layer in NON_disinfection_ reactor; (e) black layer in UV_disinfection_ reactor; (f) yellow layer in UV_disinfection_ reactor. Magnification = 5000 ×

**Figure S5** Elemental composition of the corrosion scales at 52nd week of (a) black layer in NaClO_disinfection_ reactor; (b) yellow layer in NaClO_disinfection_ reactor; (c) black layer in NON_disinfection_ reactor; (d) yellow layer in NON_disinfection_ reactor; (e) black layer in UV_disinfection_ reactor; (f) yellow layer in UV_disinfection_ reactor

**Figure S6** XRD spectrogram of cast iron corrosion products of black layer in (a) NaClO_disinfection_ reactor at 4th week; (b) NON_disinfection_ reactor at 4th week; (c) UV_disinfection_ reactor at 4th week; (d) NaClO_disinfection_ reactor at 34th week; (e) NON_disinfection_ reactor at 34th week; (f) UV_disinfection_ reactor at 34th week

**Figure S7** XRD spectrogram of cast iron corrosion products of yellow layer in (a) NaClO_disinfection_ reactor at 4th week; (b) NON_disinfection_ reactor at 4th week; (c) UV_disinfection_ reactor at 4th week; (d) NaClO_disinfection_ reactor at 34th week; (e) NON_disinfection_ reactor at 34th week; (f) UV_disinfection_ reactor at 34th week

**Figure S8** Cell counts of dead, live and total bacteria in black layer (a, b, c) and yellow layer (d, e, f). In the legends, B represents the black layer and Y represents the yellow layer, respectively. Each data point represents average cell numbers of the black layer or the yellow layer for three pieces of cast iron coupons (n=3). Error bars represent the standard deviation. The red dashed lines were used to indicate the time segmentation corresponding to the quantity change trend of viable microbes in the blank layer and yellow layer, respectively.

**Figure S9** Microbial activity in (a) black layer and (b) yellow layer under different disinfection conditions. In the legends, B represents the black layer and Y represents the yellow layer, respectively. Each data point represents the average ATP of the black layer or the yellow layer for three pieces of cast iron coupons (n=3) Error bars represent the standard deviation. The red dashed lines were used to indicate the time segmentation corresponding to the ATP variation trend in the blank layer and yellow layer, respectively.

**Figure S10** Diversity index expressed as (a) Chao1 index, (b) Shannon index and (c) Simpson index. Evenness is expressed by Evenness index (d). In the legends, B represents the black layer and Y represents the yellow layer, respectively.

**Figure S11** Community composition of black and yellow layer in three reactors were presented in the ordination space of PCoA based on weighted UniFrac distance. The numbers next to the symbols represent the week in which the sample was taken. In the legends, B represents the black layer and Y represents the yellow layer, respectively.

**Figure S12** Abundances of different phyla in 120 samples including yellow and black layers under different disinfection conditions. The number corresponding to each column represents the week in which the sample was taken.

**Figure S13** Heat map shows the total abundance distribution of top 50 genera in all black and yellow samples. The number corresponding to each column represents the week in which the sample was taken.

**Figure S14** Extended error bar plots showing abundance of genera differing significantly between NaClO_disinfection_ and UV_disinfection_ reactors with an effect size of 0.75: (a) Genera in black layer of Stage II; (b) Genera in black layer of Stage III;. (c) Genera in yellow layer of Stage II and (d) Genera in yellow layer of Stage III. In the legends, B represents the black layer and Y represents the yellow layer, respectively. The numbers in the parentheses represent the amounts of OTUs belonging to the genus correspondingly to Fig. 4. The red numbers represent the AP-type OTUs; the orange numbers represent the RP-type OTUs; and the light purple numbers represent the RI-type OTUs.

**Figure S15** The abundance variation of functional genera. (a) *Iron-oxidizing bacteria,* (b) *Iron-reducing bacteria,* (c) *Nitrite-oxidizing bacteria,* (d) *Nitrate-reducing bacteria,* (e) *Sediminibacterium*; (f) *Geobacter*. In the legends, B represents the black layer; and the Y represents the yellow layer, respectively. The numbers in the parentheses represent the amounts of OTUs belonging to the category/genus correspondingly to Fig. 4. The red numbers represent the AP-type OTUs; the orange numbers represent the RP-type OTUs; and the light purple numbers represent the RI-type OTUs.

**Text S1: Detection method summary of water quality**

Dissolved oxygen (DO), pH, conductivity and oxidation-reduction potential (ORP) were measured using portable multi-parameter meter (sensION156 HACH, USA).

The concentration of Ca, Mg and Fe ions were analyzed by the Inductively Coupled Plasma Optical Emission Spectrometer (PerkineElmer Optima 2000, USA) according to “Analysis Method for Water and Wastewater” which was issued by Ministry of Environmental Protection, China.

Total nitrogen (TN) was determined by alkaline potassium persulphate digestion-UV spectrophotometric method using HACH DR3900 spectrophotometer (USA) following “Analysis Method for Water and Wastewater”, which was issued by Ministry of Environmental Protection, China.

Total phosphorus (TP) was determined by ammonium molybdate spectrophotometry method using HACH DR3900 spectrophotometer (USA) following “Analysis Method for Water and Wastewater”*,* which was issued by Ministry of Environmental Protection, China.

Free chlorine and total chlorine were determined according to the Methods 8167 and 8021 in “Water Analysis Handbook” using the HACH DR3900 spectrophotometer (USA) and the corresponding commercial kits (Cat. 2105569 and Cat. 2105669) purchased from HACH company.

Total organic carbon (TOC) was determined using TOC-L (SHIMADZU, Japan) adopting the 680 ℃ combustion catalytic oxidation method following “Analysis Method for Water and Wastewater”, which was issued by Ministry of Environmental Protection, China.

**Text S2: Evaluation of the effect of sonication pretreatment on the bacterial cell viability and ATP measurement**

Ultrasonic approach with high frequencies, i.e., 42 kHz, 5 min × 3 times was used to detach bacteria from the black layer as much as possible. Sonication pretreatment used in this study might affect the viability of bacterial cells, ATP measurement and thus lead to biased results. To evaluate the possible bias of sonication on bacterial viability and ATP results, a two-month preliminary experiment was conducted before the formal experiment. Three pieces of cast iron coupons collected from NON_disinfection_ reactor at 8th week were used as triplicates to estimate the effect of ultrasonic on microbial activity and cell integrity. The yellow layer of the three cast iron pieces was removed via slight flushing with ultrapure water and the black layer was acquired by blade scraping. The details of the subsequent experiment were showed in the following flowchart.

The viable and dead bacteria in samples of S_0_-S_4_ were measured using flow cytometry and the ATP measurement was conducted using the BacTiter-Glo^TM^ reagent (Promega Corporation, Madison, USA) and a luminometer (SpectraMax i3, Molecular Devices, USA). The detailed procedures were the same as the corresponding description in Methods section of the manuscript. The ratio of dead cell number to total cell number and the ATP results are summarized in the following table.

| Sample ID | Dead cell number/Total cell number (%) | ATP (nmol/cm^2^) |
| --- | --- | --- |
| S_0_  (before ultrasonication) | 40.4±2.2 | 20.9±1.9 |
| S_1_  (Round 1 ultrasonication) | 41.4±1.0 | 20.7±0.7 |
| S_2_  (Round 2 ultrasonication) | 44.0±2.4 | 20.4±1.9 |
| S_3_  (Round 3 ultrasonication) | 43.9±1.8 | 18.8±1.6 |

These results indicated that the ultrasonic pretreatment process used in the present study did not affect the viability of bacterial cells and ATP measurement.

.

**Table S1** Water quality parameters of NaClO_disinfection_ reclaimed wastewater, NON_disinfection_ reclaimed wastewater and UV_disinfection_ reclaimed wastewater

| Water quality parameter | NaClO_disinfection_ reclaimed wastewater (5mg/L) | NON_disinfection_ reclaimed wastewater | UV_disinfection_ reclaimed wastewater (27mJ/cm^2^) |
| --- | --- | --- | --- |
| pH | 6.67±0.16 | 6.62±0.13 | 6.64±0.12 |
| ORP (mv) | 480.2±83.6 | 290.8±87.1 | 282.0±80.4 |
| Conductivity (μS cm^-1^) | 473.2±62.9 | 425.8±57.1 | 426.0±54.6 |
| Mg (mg/L) | 4.04±0.94 | 4.05±1.05 | 4.07±0.92 |
| Ca (mg/L) | 34.9±3.5 | 34.9±3.3 | 34.9±3.3 |
| DO (mg/L) | 3.47±0.99 | 3.63±1.05 | 4.47±0.75 |
| Free-Cl(mg/L) | 0.44±0.49 | 0.00±0.00 | 0.00±0.00 |
| Total-Cl (mg/L) | 1.31±0.93 | 0.00±0.00 | 0.00±0.00 |
| TN (mg/L) | 10.9±2.4 | 11.2±2.2 | 11.1±2.0 |
| Fe (mg/L) | 0.36±0.16 | 0.34±0.16 | 0.33±0.14 |
| TP (mg/L) | 0.16±0.10 | 0.15±0.10 | 0.16±0.11 |
| TOC (mg/L) | 5.44±1.41 | 4.74±1.48 | 4.54±1.22 |

**Table S2** Sampling time points

| Sampling week |  | Sampling date |
| --- | --- | --- |
| 1st week |  | 2016-11-20 |
| 2nd week |  | 2016-11-27 |
| 3rd week |  | 2016-12-04 |
| 4th week |  | 2016-12-11 |
| 7th week |  | 2017-01-01 |
| 10th week |  | 2017-01-22 |
| 13th week |  | 2017-02-12 |
| 16th week |  | 2017-03-05 |
| 19th week |  | 2017-03-26 |
| 22nd week |  | 2017-04-16 |
| 25th week |  | 2017-05-07 |
| 28th week |  | 2017-05-28 |
| 31st week |  | 2017-06-18 |
| 34th week |  | 2017-07-09 |
| 37th week |  | 2017-07-30 |
| 40th week |  | 2017-08-20 |
| 43rd week |  | 2017-09-10 |
| 46th week |  | 2017-10-01 |
| 49th week |  | 2017-10-22 |
| 52nd week |  | 2017-11-12 |


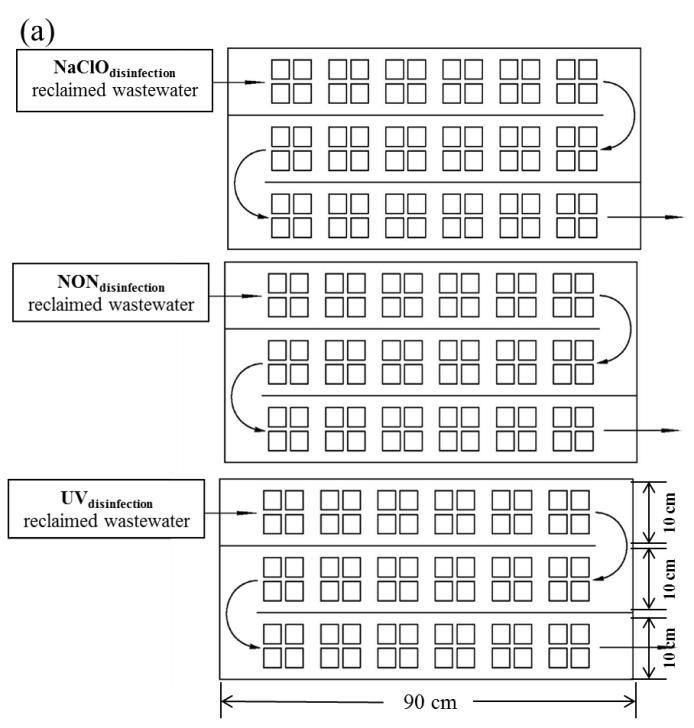

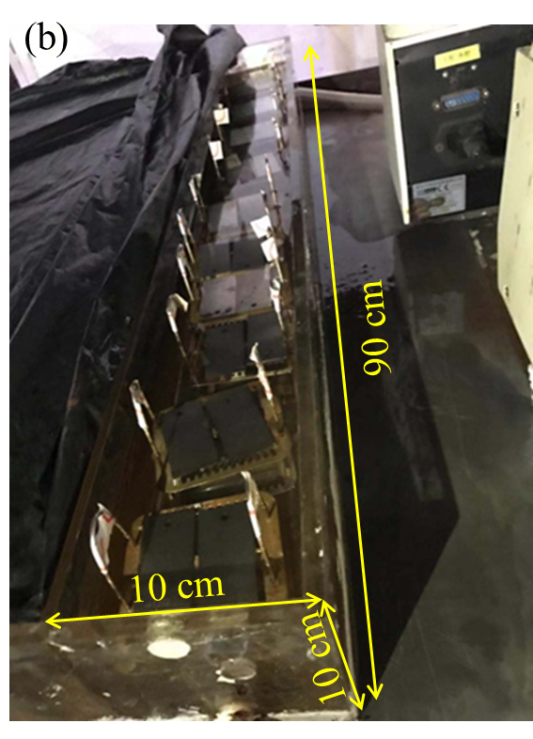


**Figure S1** (a) Schematic experimental setup used for laboratory-scale study. The effective size of each reactor is 90 cm×30 cm×10 cm (length×width×depth). There are three lanes in each reactor with the effective size of 90 cm×30 cm×10 cm (length×width×depth).The arrow represents the water flow direction and the flow rate was 0.2 m/s. (b) The actual picture showing one lane (90 cm×30 cm×10 cm) of the NaClO_disinfection_ reactor.


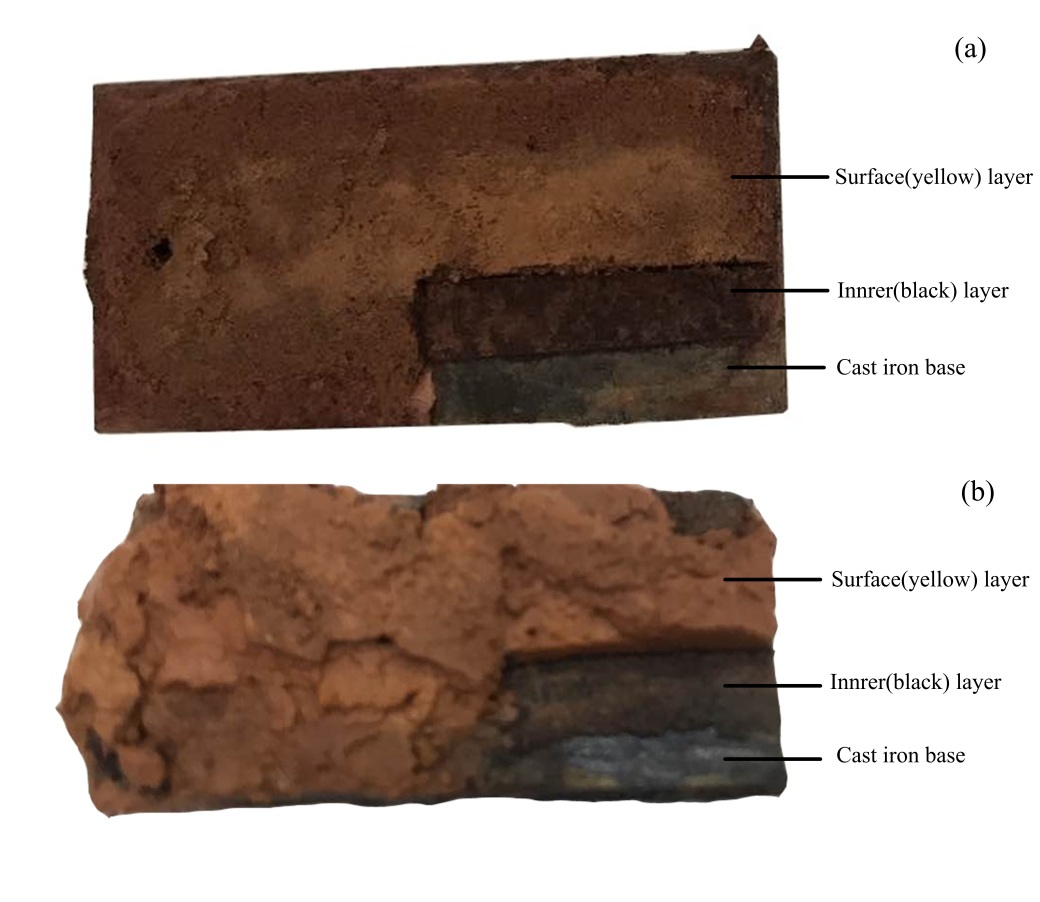


**Figure S2** Surface (yellow) layer and inner (black) layer were defined according to its color. (a) Cast iron coupon in NaClO_disinfection_ reactor at 52nd week. The thickness of the yellow layer and black layer is approximately 2.0~2.5 mm and 0.5~1 mm, respectively; (b) Cast iron coupon in NON_disinfection_ reactor at 52nd week. The thickness of the yellow layer and black layer is approximately 7.5~9.5 mm and 2.0~3.0 mm, respectively. The thickness of the underlying cast iron bases in the NaClO_disinfection_ reactor and the NON_disinfection_ reactor were 1.71-1.75 mm and 1.60-1.63 mm, respectively.


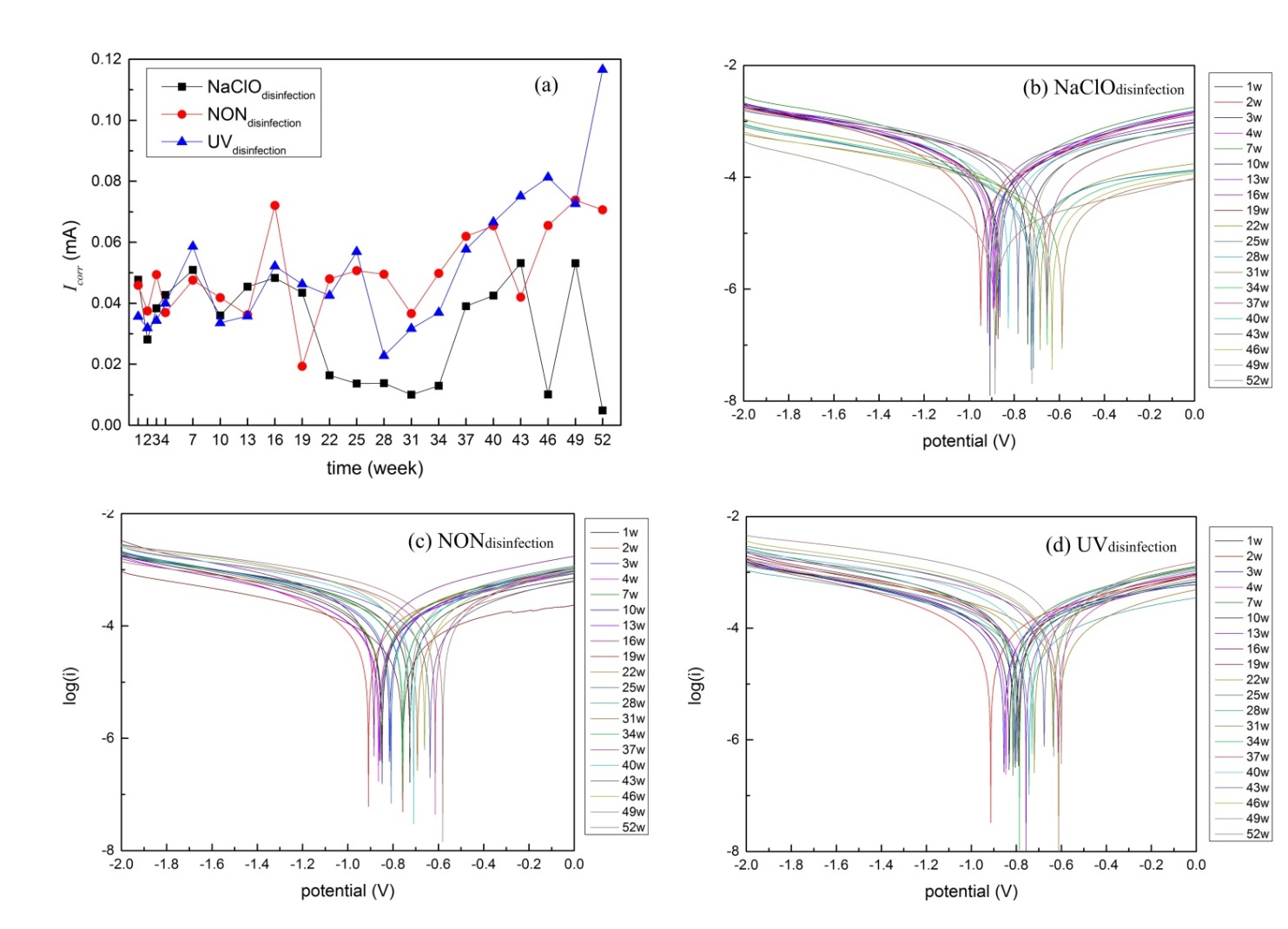


**Figure S3** (a) The corrosion current of cast iron coupons in NaClO _disinfection_, UV _disinfection_ and NON _disinfection_ reactors over one year; Polarization curves of cast iron coupons in (b) NaClO_disinfection_ reactor, (c) NON _disinfection_ reactor and (d) UV _disinfection_ reactor. Data points indicates n=1, curves indicate n=1.

**Figure S4** SEM micrograph of the cast iron corrosion scale at 52nd week. (a) black layer in NaClO_disinfection_ reactor; (b) yellow layer in NaClO_disinfection_ reactor; (c) black layer in NON_disinfection_ reactor; (d) yellow layer in NON_disinfection_ reactor; (e) black layer in UV_disinfection_ reactor; (f) yellow layer in UV_disinfection_ reactor. Magnification = 5000×.

**Figure S5** Elemental composition of the corrosion scales at 52nd week of (a) black layer in NaClO_disinfection_ reactor; (b) yellow layer in NaClO_disinfection_ reactor; (c) black layer in NON_disinfection_ reactor; (d) yellow layer in NON_disinfection_ reactor; (e) black layer in UV_disinfection_ reactor; (f) yellow layer in UV_disinfection_ reactor

**Figure S6** XRD spectrogram of cast iron corrosion products of black layer in (a) NaClO_disinfection_ reactor at 4th week; (b) NON_disinfection_ reactor at 4th week; (c) UV_disinfection_ reactor at 4th week; (d) NaClO_disinfection_ reactor at 34th week; (e) NON_disinfection_ reactor at 34th week; (f) UV_disinfection_ reactor at 34th week

**Figure S7** XRD spectrogram of cast iron corrosion products of yellow layer in (a) NaClO_disinfection_ reactor at 4th week; (b) NON_disinfection_ reactor at 4th week; (c) UV_disinfection_ reactor at 4th week; (d) NaClO_disinfection_ reactor at 34th week; (e) NON_disinfection_ reactor at 34th week; (f) UV_disinfection_ reactor at 34th week

**
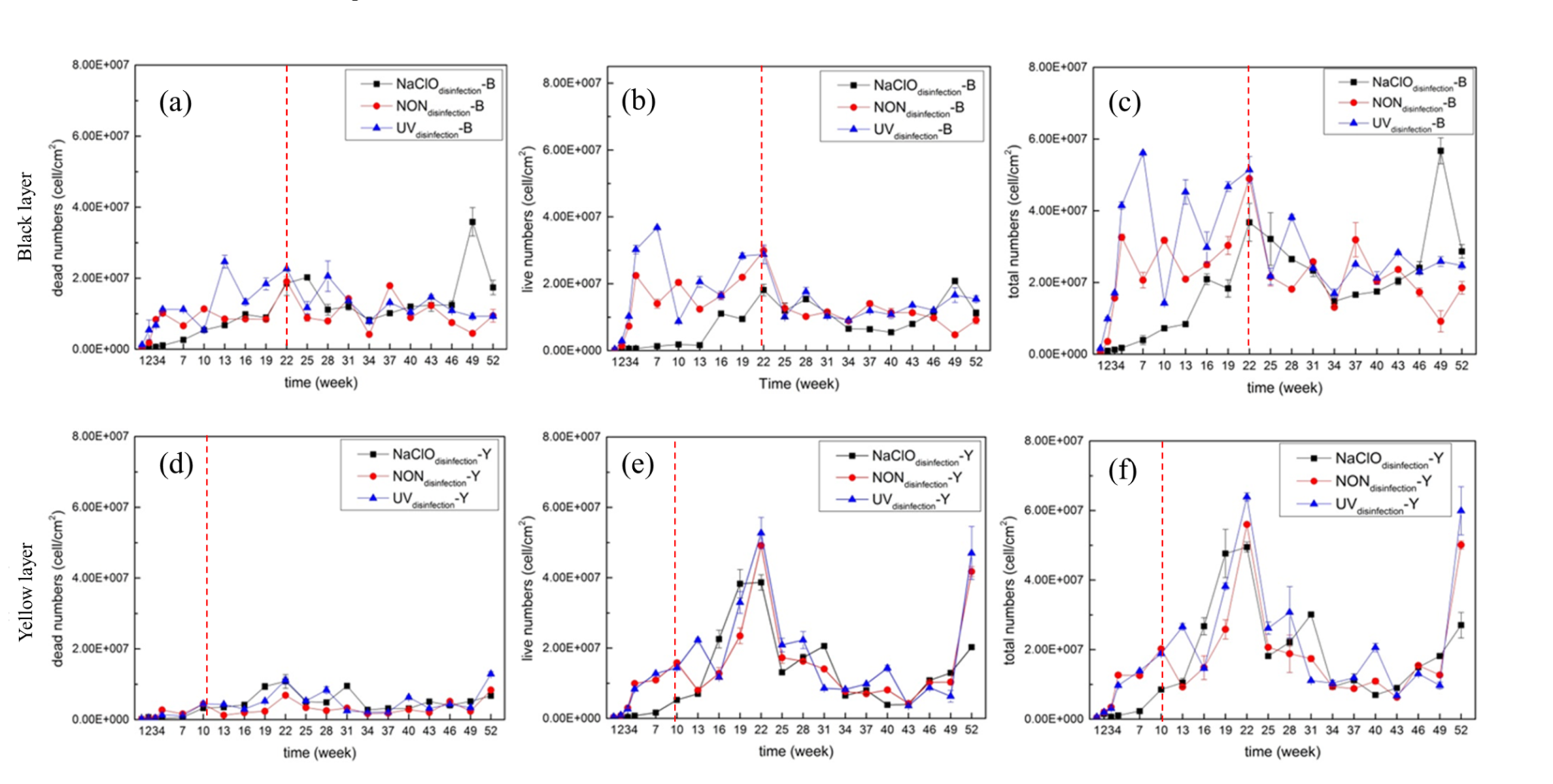
**

**Figure S8** Cell counts of dead, live and total bacteria in black layer (a, b, c) and yellow layer (d, e, f). In the legends, B represents the black layer and Y represents the yellow layer, respectively. Each data point represents average cell numbers of the black layer or the yellow layer for three pieces of cast iron coupons (n=3). Error bars represent the standard deviation. The red dashed lines were used to indicate the time segmentation corresponding to the quantity change trend of viable microbes in the blank layer and yellow layer, respectively.


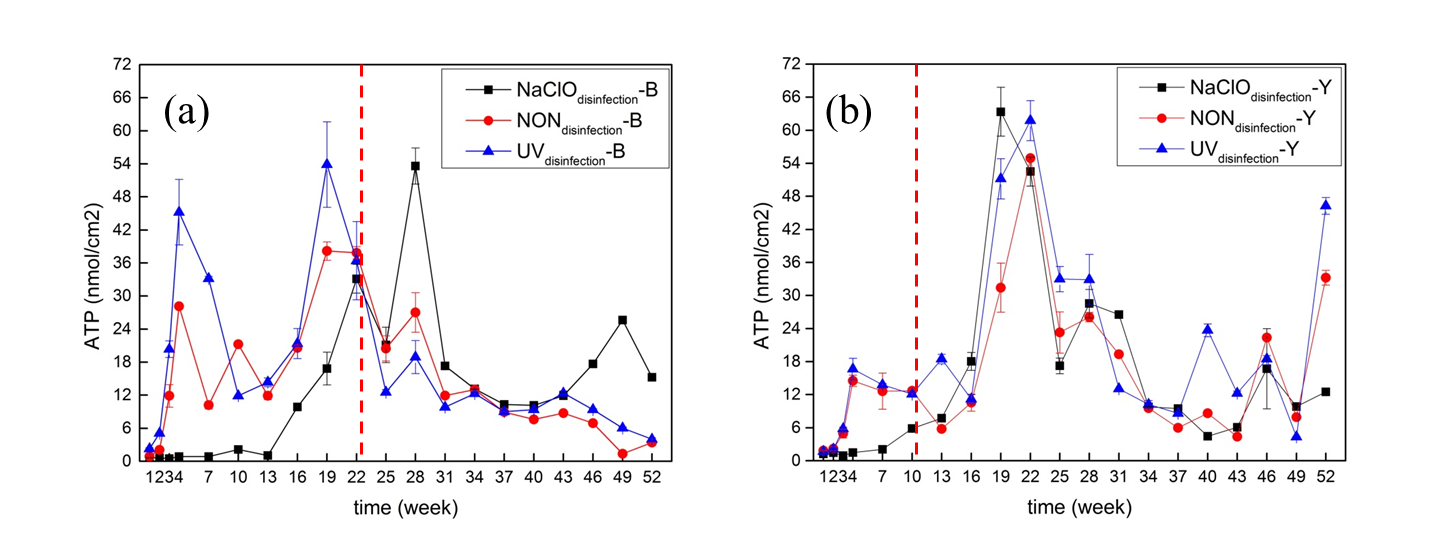


**Figure S9** Microbial activity in (a) black layer and (b) yellow layer under different disinfection conditions. In the legends, B represents the black layer and Y represents the yellow layer, respectively. Each data point represents the average ATP of the black layer or the yellow layer for three pieces of cast iron coupons (n=3) Error bars represent the standard deviation. The red dashed lines were used to indicate the time segmentation corresponding to the ATP variation trend in the blank layer and yellow layer, respectively.


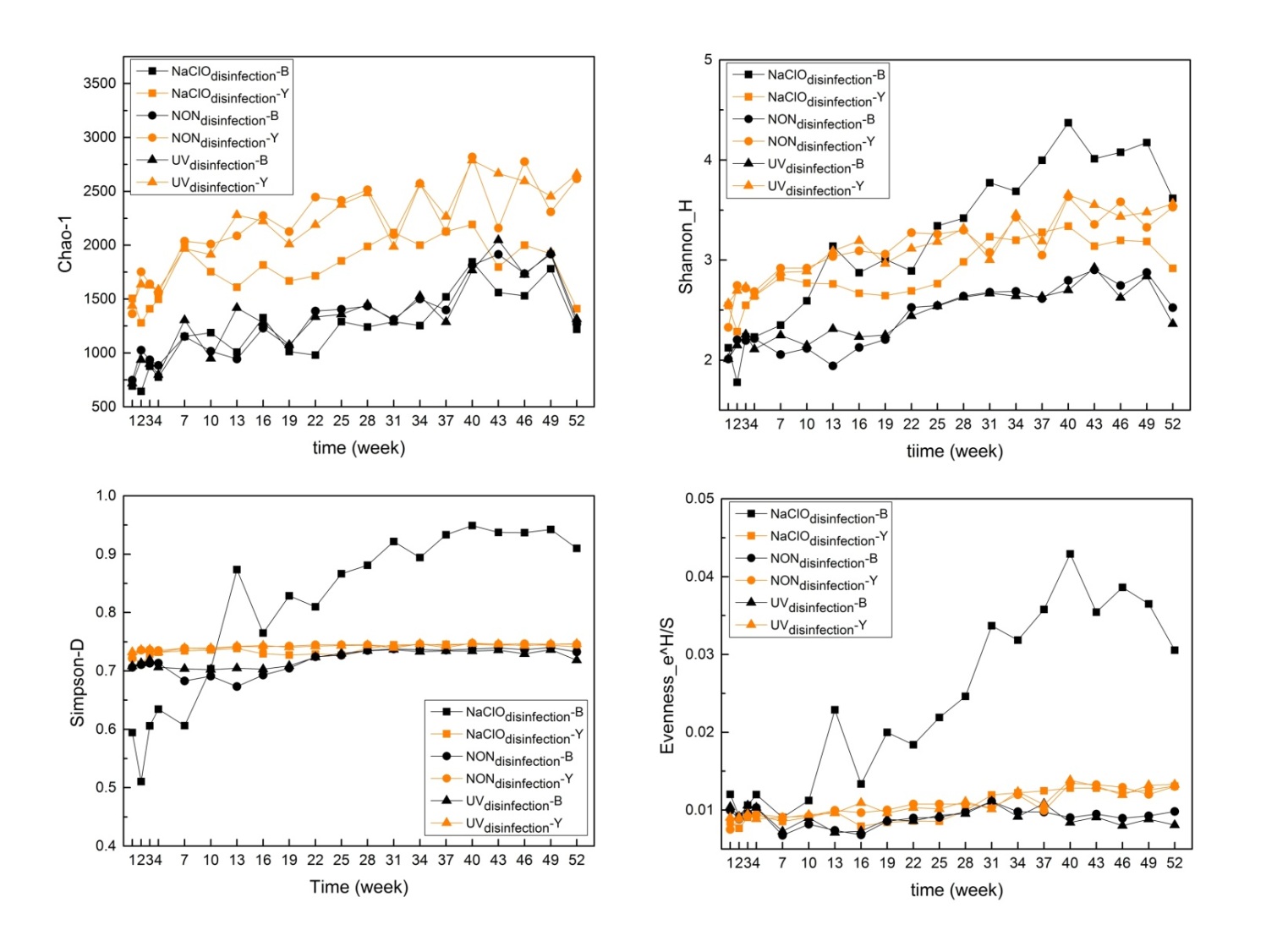


(d)

(c)

(b)

(a)

**Figure S10** Diversity index expressed as (a) Chao1 index, (b) Shannon index and (c) Simpson index. Evenness is expressed by Evenness index (d). In the legends, B represents the black layer and Y represents the yellow layer, respectively.

**Figure S11** Community composition of black and yellow layer in three reactors were presented in the ordination space of PCoA based on weighted UniFrac distance. The numbers next to the symbols represent the week in which the sample was taken. In the legends, B represents the black layer and Y represents the yellow layer, respectively.

**Figure S12** Abundances of different phyla in 120 samples including yellow and black layers under different disinfection conditions. The number corresponding to each column represents the week in which the sample was taken.

**Figure S13** Heat map shows the total abundance distribution of top 50 genera in all black and yellow samples. The number corresponding to each column represents the week in which the sample was taken.


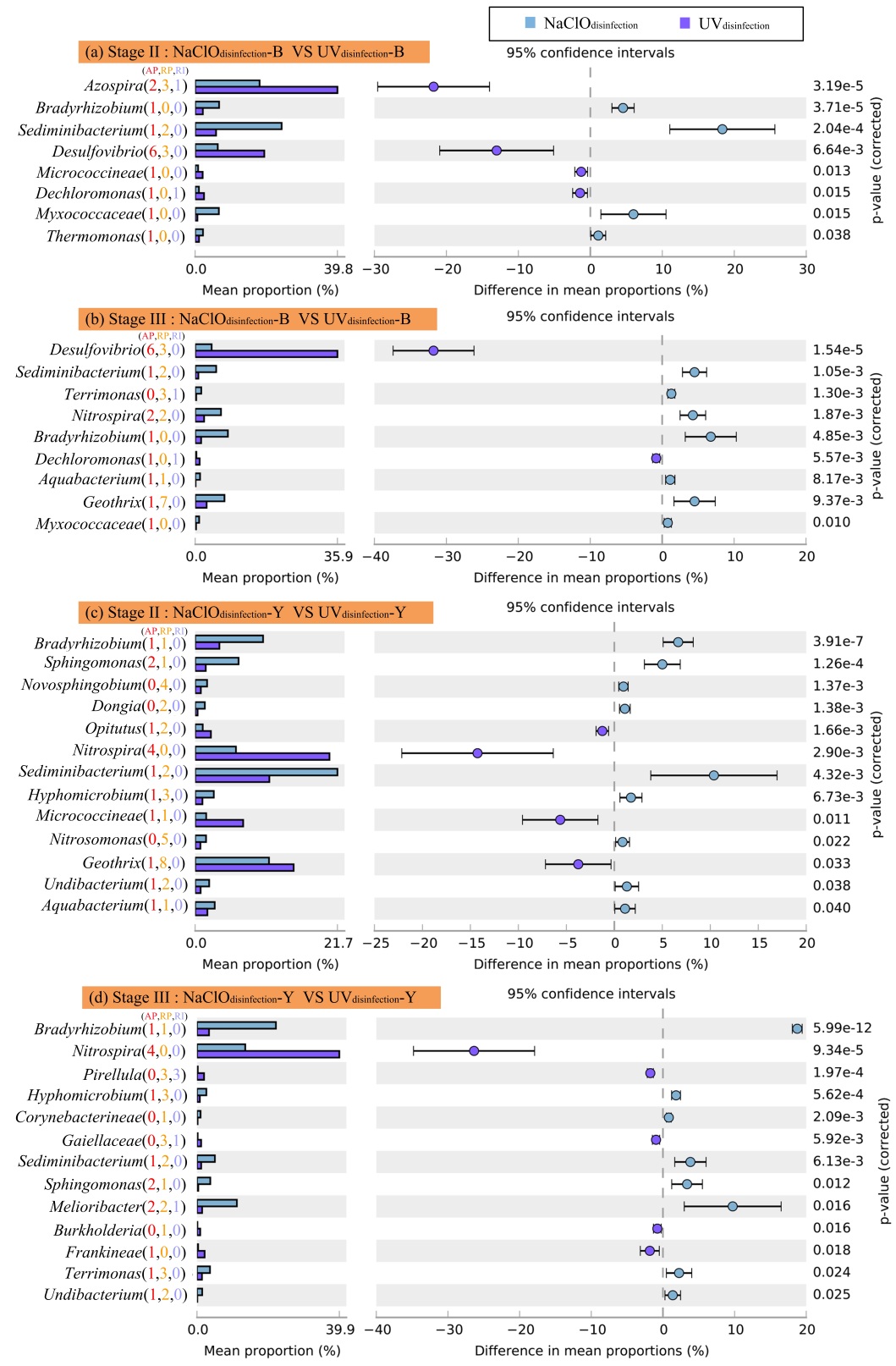


**Figure S14** Extended error bar plots showing abundance of genera differing significantly between NaClO_disinfection_ and UV_disinfection_ reactors with an effect size of 0.75: (a) Genera in black layer of Stage II; (b) Genera in black layer of Stage III;. (c) Genera in yellow layer of Stage II and (d) Genera in yellow layer of Stage III. In the legends, B represents the black layer and Y represents the yellow layer, respectively. The numbers in the parentheses represent the amounts of OTUs belonging to the genus correspondingly to Fig. 4. The red numbers represent the AP-type OTUs; the orange numbers represent the RP-type OTUs; and the light purple numbers represent the RI-type OTUs.


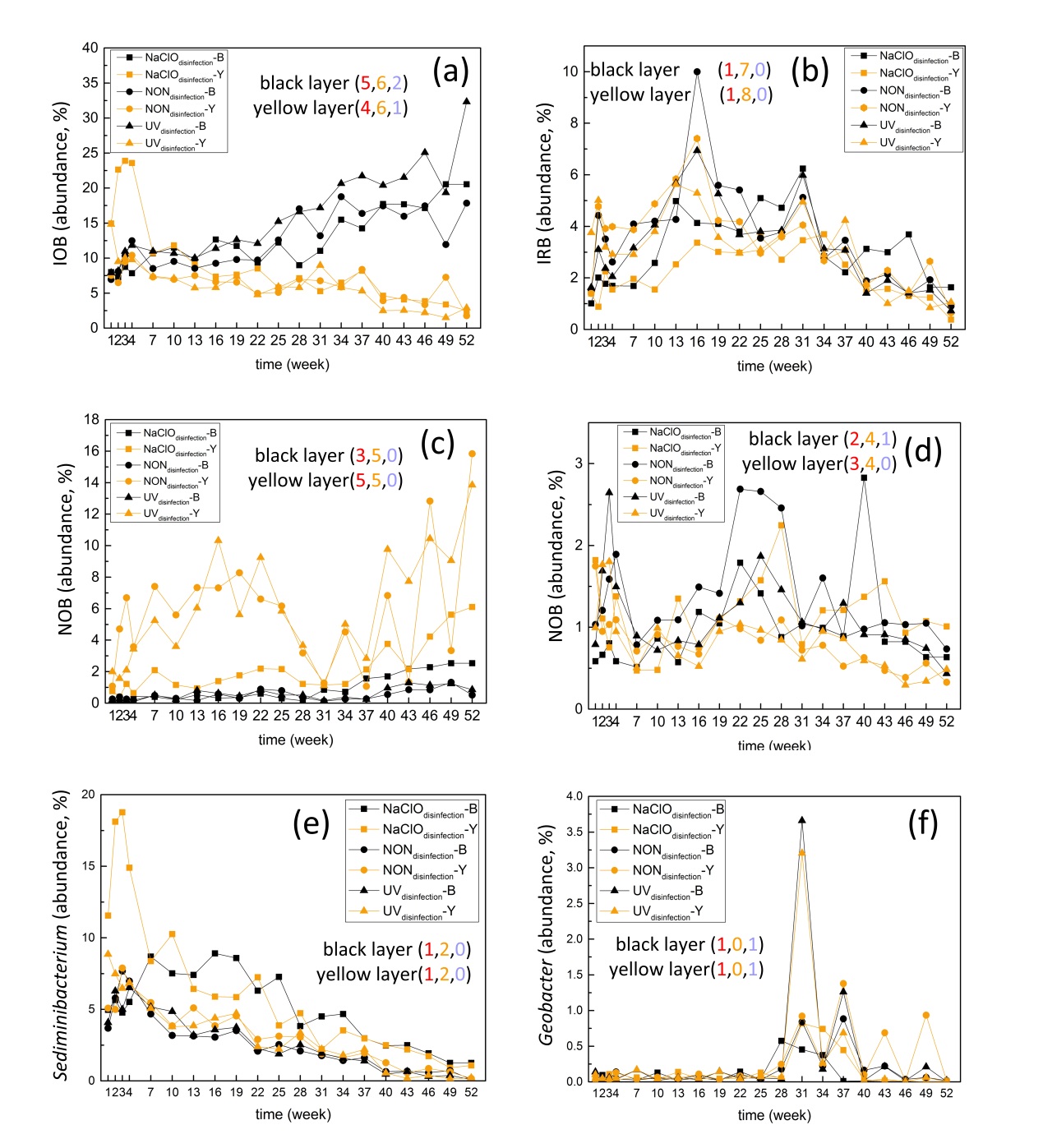


**Figure S15** The abundance variation of functional genera. (a) *Iron-oxidizing bacteria,* (b) *Iron-reducing bacteria,* (c) *Nitrite-oxidizing bacteria,* (d) *Nitrate-reducing bacteria,* (e) *Sediminibacterium*; (f) *Geobacter*. In the legends, B represents the black layer and Y represents the yellow layer, respectively. The numbers in the parentheses represent the amounts of OTUs belonging to the category/genus correspondingly to Fig. 4. The red numbers represent the AP-type OTUs; the orange numbers represent the RP-type OTUs; and the light purple numbers represent the RI-type OTUs.
